# Somatostatin in Aging: Correlations with Selected Central Nervous System and Gastrointestinal Tract Diseases

**DOI:** 10.3390/ijms27104244

**Published:** 2026-05-10

**Authors:** Aldona Kasprzak

**Affiliations:** Department of Histology and Embryology, Swiecicki Street 6, Poznan University of Medical Sciences, 60-781 Poznań, Poland; akasprza@ump.edu.pl; Tel.: +48-61-8546441

**Keywords:** somatostatin, aging, CNS, GI tract, Alzheimer’s disease, Parkinson’s disease, obesity, diabetes mellitus, colorectal cancer, somatostatin-based therapy in age-related diseases

## Abstract

The hypothalamic–pituitary–somatotropic (HPS) axis, which includes growth hormone (GH) and insulin-like growth factor 1 (IGF-1), is one of three endocrine systems that show a decline in hormone concentration with age. Among the hypothalamic hormones involved in the aging process, GH-releasing hormone (GHRH) and somatostatin (SST) are most affected, resulting in several age-related changes. The pathophysiology of GH decline in the aging process is unclear, specifically, whether it results from decreased GHRH or increased SST levels. Similarly, it is not known whether quantitative changes in hypothalamic peptides (including SST) precede or follow age-related pathological behavioral changes. SST is produced mainly by cells of the central nervous system (CNS) and the gastrointestinal (GI) tract, which are functionally interconnected systems that undergo significant changes during aging. The physical changes in the aging organism are considered physiological, and experimental evidence indicates that a large proportion of these changes are the result of declining hormonal activity (including the SST system). It is particularly important to understand the role of SST in diseases of old age, which affect both cognitive processes and memory (e.g., Alzheimer’s and Parkinson’s diseases) and the proper functioning of the GI tract and pancreas (e.g., obesity, type 2 diabetes mellitus, and colorectal cancer). This narrative review discusses systemic and peripheral changes in SST production and secretion observed in aging individuals and their potential association with selected diseases of old age, especially CNS and GI tract diseases. Understanding the role of SST expression with age will enable the better application of this neuropeptide in the diagnosis and treatment of diseases of old age (including cancers).

## 1. Introduction

The tetradecapeptide somatostatin (SST) with the sequence H-Ala-Gly-Cys-Lys-Asn-Phe-Phe-Trp-Lys-Thr-Phe-Trh-Ser-Cys-OH was first isolated from the sheep hypothalamus. At a concentration of 1 × 10^−9^ M, it inhibited the secretion of growth hormone (GH, somatotropin) in vitro (rats/humans) and showed similar activity in vivo (rats) [1], thus the name somatotropin/GH release-inhibiting factor/hormone (SRIF/SRIH or GHIRH). Subsequent isolation and sequencing of the human *SST* gene [2] allowed for the study of the peptide’s properties and the regulation of its gene expression in non-hypothalamic organs, including the gastrointestinal (GI) tract [3,4,5,6]. The human *SST* gene is located on chromosome 3 and has 1 transcript (splice variant) and 262 orthologues. It contains a single intron that interrupts the coding sequence in the propeptide region of the SST molecule [2,7]. SST expression in cells is regulated before translation by methylation and polymorphisms in the promoter region, as well as by the activity of various transcription factors (reviewed in [8]). The SST peptide occurs in two biologically active forms: a 14 amino acid (AA) form (SST-14) and a 28 AA form (SST-28), derived from a larger precursor, preprosomatostatin, approximately 120 AA in length [8,9]. SST-14 is secreted mainly by nerve cells and pancreatic islet δ cells, while SST-28 is the main product of D cells in the GI tract [10]. In the mammalian brain, the SST family consists of two peptides, SST and cortistatin (CST), which interact with five receptor subtypes (SST1-5, SSTRs) that belong to the G protein-coupled receptor (GPCR) family [7]. CST was cloned in 1996 and shares 11 AA with SST [11,12].

The hypothalamic–pituitary–somatotropic (HPS) axis, which includes GH and insulin-like growth factor-1 (IGF-1), is one of three endocrine systems that show a decline in hormone concentration with age. GH secretion is inhibited by IGF-1 through a feedback loop, as well as by SST and other neuroendocrine signals, including insulin, which act by binding to their respective receptors (reviewed in [13]). Disturbances in the entire HPS system result in many of the catabolic consequences of the normal aging process [14,15,16,17,18]. On the other hand, the physiological changes that occur in the human body during aging are similar to those observed in GH deficiency (GHD) [14,17,19,20]. The pathophysiology of GH decline in the aging process is unclear; it may result from decreased GH-releasing hormone (GHRH) or increased SST levels [15,16,21].

For many human GI tract cancers (e.g., stomach, liver, pancreas, and colorectal), the key risk factors are the patient’s age and age-related pathological conditions (e.g., overweight and obesity, diabetes, and chronic intestinal inflammation) [22,23,24,25,26,27,28]. Although the mechanisms of aging and carcinogenesis differ, common phenomena in both processes include the accumulation of DNA damage and abnormal proteins, a shift in the stromal environment, and weakened immune function [22,28,29].

Variable expression of SST and/or SST1-5 has been confirmed, particularly in numerous neuroendocrine tumors/neoplasms (NETs/NENs) in humans across different locations [30,31,32,33,34]. It should be noted that, as with NETs, the highest increase in incidence occurs among older people aged ≥65 years [35]. Particularly rich in SST expression and its receptors are (gastro)pancreatic (GEP) NETs [36,37,38]. SST expression occurs not only in sporadic NETs of the duodenum and pancreas but may also occur in gangliocytic paragangliomas (GCPGs), poorly differentiated neuroendocrine carcinomas (pdNEC), and hereditary NETs. SST-NET and GCPG associated with multiple endocrine neoplasia type 1 (MEN1) have a benign course, while SST-producing pdNEC are malignant tumors. The occurrence of the so-called somatostatinoma syndrome is extremely rare [36]. Variable expression of SST system components has also been demonstrated in sporadic non-neuroendocrine colorectal cancer (CRC) [39,40,41,42].

Due to the presence of several SST production sites and complex functions in the central nervous system (CNS) and peripheral tissues, research on the role of this neuropeptide (NP) is ongoing, especially regarding its clinical and diagnostic-therapeutic implications [6,8,27,35,43]. It should be emphasized that SST is produced in both the CNS and the GI tract, functionally interconnected systems [27] that undergo significant changes during aging.

This narrative review discusses systemic and peripheral changes in SST production and secretion observed in aging individuals and their potential association with selected diseases of old age, especially CNS and GI tract diseases. Understanding the role of SST expression with age will enable better application of this neuropeptide in the diagnosis and treatment of diseases of old age (including cancers).

A literature search was performed in PubMed to identify relevant articles published within January 1980 to December 2025. The following search (MeSH) terms we used: “somatostatin”, “aging”/“age-related changes”, “central nervous system”, “gastrointestinal tract”, “Alzheimer’s disease”, “Parkinsons’disease”, “obesity”, “type 2 diabetes mellitus”, “colorectal cancer”. In addition, the term “aging and somatostatin” has been linked to all the other keywords. The inclusion criteria for the selection of articles were as follows: (1) studies published in peer-reviewed journals; (2) articles available in English, and (3) seminal papers considered essential for the review, including articles from 2026.

## 2. The Endocrine System in Aging

Among the molecular features of aging defined in 2013 were genomic instability, telomere attrition, epigenetic alterations, loss of proteostasis, deregulated nutrient sensing, mitochondrial dysfunction, cellular senescence, stem cell (SC) exhaustion, and altered intercellular communication [44]. Three new features were added to the version updated 10 years later, namely: disabled macroautophagy, chronic inflammation, and dysbiosis [45]. In general, there are three categories of aging characteristics: (1) molecular characteristics (listed above); (2) cellular features (cell aging, SC depletion, and altered intercellular communication); and (3) systemic changes (impaired nutrient regulation) [46]. In relation to cellular senescence, this is a state of permanent cell cycle arrest caused by various stress factors (e.g., DNA damage, endoplasmic reticulum (ER) stress, and oncogene activation) [47,48,49]. Complete growth arrest is associated with the upregulation of various cell cycle inhibitors, mainly p16 and p21, structural and metabolic changes, chronic responses to DNA damage, and a state of excessive secretion known as the senescence-associated secretory phenotype (SASP) [47,50,51].

The aging process is accompanied by the accumulation of aging cells and progressive metabolic changes. Modification of N6-methyladenosine (m6A) methylation regulates the aging process of cells and tissues by modulating senescence-related genes. Key signaling pathways associated with aging include the insulin/IGF-1, the mammalian target of rapamycin (mTOR), adenosine monophosphate-activated protein kinase (AMPK), nuclear factor kappa-light-chain-enhancer of activated B cells (NF-kB) and sirtuin pathways. These pathways regulate glucose, amino acids, cyclic adenosine monophosphate (cAMP), and nicotinamide adenine dinucleotide(+) (NAD(+)) levels by absorbing nutrients or metabolic products, forming complex networks related to longevity and aging (reviewed in [48]).

Although the characteristics of cell aging in vitro are fairly well understood, the mechanisms of aging in living organisms are less well-known [52]. There are numerous theories of aging and the characteristics of age-related gene expression, as well as various therapeutic strategies related to diseases in older people [53,54] and animals [55]. Genome-wide gene expression profiling of the human brain has identified genes whose expression changes throughout life. Strong age-related upregulation of genes that are highly expressed in oligodendrocytes and astrocytes was discovered, while genes highly expressed in layer 2/3 glutamatergic neurons were downregulated throughout life. Analysis of genes enriched in a given cell type, based on gene ontology, highlighted a strong downregulation of genes involved in synaptic transmission and intercellular signaling in the SST neuron subtype, which expresses cyclin-dependent kinase 6 (Cdk6), and in the vasoactive intestinal peptide (VIP) neuron subtype, which expresses myosin-binding protein C, slow-type (Mybpc1). These findings provide a number of original insights into the specific vulnerability of cells to normal aging. Additionally, they suggest age-related synaptic changes in specific subtypes of inhibitory neurons [56].

The mechanisms of endocrine dysfunction due to aging are tissue- and species-specific. The dominant mechanism in each age-related change varies depending on the hormone being studied [57,58]. At the molecular level, endocrine gland aging is closely linked to oxidative stress, cellular autophagy, genetic damage, and hormone secretion. Considering the order in which endocrine organs age, the hypothalamus and pituitary gland rank second and third, respectively, after the pineal gland (reviewed in [59]). The aging process affects the complex interactions between the hormones of the hypothalamus and pituitary gland. Most of these systems become less sensitive with age, due to reduced function of peptide-containing secretory neurons, loss of hormone receptor sensitivity, and/or decreased efficiency of target endocrine glands [55,60].

Anatomical changes in the endocrine glands may occur, in particular, as a result of programmed cell death, autoimmune destruction, or malignant transformation of the gland. Age-related changes in hormone secretion may also be secondary to physiological changes in circadian and seasonal rhythms or in the frequency or amplitude of hormonal pulses. The bioactivity of hormones, hormone transport to receptor binding sites, hormone–receptor interactions, and post-receptor effects may also be altered. In light of these latter effects, aging is associated with changes in the characteristics of cell membranes, alterations in the activity of cellular enzymes, and changes in calcium mobilization and gene expression. Some of these changes are directly related to aging, while others are secondary to age-related diseases and changes in nutritional status (reviewed in [55]).

There is considerable evidence to support the theory that the aging process is regulated by the hypothalamus through its role in maintaining energy homeostasis, hormonal regulation, circadian rhythms, and reproductive functions via age-related changes in key neural regulators. Among the hypothalamic hormones involved in the aging process, GHRH and SST are affected, resulting in several age-related changes. GH is a powerful metabolic hormone, often touted as the “fountain of youth.” It has many targets throughout the body, mainly in the brain, liver, and muscles (reviewed in [61]).

The gradual decline in GH secretion in humans begins at around the age of 30, with the greatest decline observed at the age of 70. The average decline is approx. 14% per decade. Research by Toogood et al. has shown that organic GHD in older people differs from the decline in GH secretion associated with the aging process [62]. Attempts have been made to explain the causes of age-related decline in GH secretion. The term ”somatopause” has been introduced to describe the age-related decline in pulsatile GH levels and the resulting decrease in blood IGF-1 concentrations [15,16,21,62,63]. Other authors equate the term “somatopause” with “somatopenia” (explaining the Greek origin of the term) [64]. It was assumed that the main cause of somatopause was reduced central cholinergic activity leading to unrestricted release of SST [19,63,64,65]. In rats [66] and humans [67], a decrease in cholinergic system stimulation has been described with age. The main neurotransmitter of this system, acetylcholine, stimulates GH release via muscarinic receptors, thereby inhibiting the action of SST neurons [66]. The decline in acetylcholine secretion with age therefore results in increased SST concentrations and inhibition of GH production in older people. Normalization of the weakened GH response to GHRH in older women using arginine would confirm this hypothesis [67]. According to other authors, the mechanisms underlying age-related GH suppression vary across species. In addition, other factors (e.g., gender, nutrition, adiposity) affect outcomes and vary across older individuals. The challenge is to study the role of SST in relation to these factors as well (reviewed in [43]). In addition, there is debate about how somatopause contributes to age-related changes in body composition, structural functions, and metabolism. Researchers continue to debate the paradox that lifelong GH/IGF-1 deficiency is associated with increased life expectancy, while GH replacement in old age may have anti-aging effects [10,13,14,19,68,69]. Although most studies in animal models have shown a link between reduced GH levels (or GH resistance) and increased lifespan, studies in humans provide conflicting results (reviewed in [70]). On the one hand, there is evidence challenging the suggestion that GHD is a beneficial adaptation to aging [68]. On the other hand, the natural decline in GH levels during aging may provide important protection against cancer and other age-related diseases [71]. It should be emphasized that elevated levels of GH and IGF-1 are associated with the development of many cancers, e.g., breast, prostate, and colorectal cancers in mammals, as most of these cancers express IGF-1 receptors [72,73]. Although animal models have demonstrated the beneficial effects of long-term treatment with physiological doses of GH on all organs and functions studied [18], it is controversial and not recommended to use GH in people with age-related GHD who do not have a known structural disease of the hypothalamus/pituitary gland. The use of GH in sport is also illegal and has no scientific or ethical basis [19,63,74].

The clinical significance of deficiencies in endocrine system components with age varies and includes reduced protein synthesis, decreased lean body mass and bone mass, increased fat mass, insulin resistance, higher risk of cardiovascular disease, worsening vasomotor symptoms, fatigue, depression, anemia, low libido, erectile dysfunction, and weakened immune function [75]. Age-related diseases primarily include: type 2 diabetes mellitus (T2DM), cardiovascular diseases, neurodegeneration, and cancers (including CRC) [24,49,52,54,76]. The pathogenesis of these diseases focuses on chronic inflammation caused by SASP [47,49,51,53,77]. Age-related disorders affect tissues that have already lost their functionality as a result of physiological aging processes, including tissues in which aging cells accumulate (so-called “primary” tissues). Aging reduces the resistance of tissues to disease-causing stresses by arresting the progenitor cell cycle and by dysfunction of stem and parenchymal cells via SASP. As the disease progresses, an additional wave of aging cells (“secondary” aging cells) forms at the sites of pathology. The latter, like “primary” cells, can exacerbate disease progression [52,53].

## 3. Somatostatin in the Central Nervous System

### 3.1. GHRH/SST Axis in Physiology

SST is one of the best-known NPs, acting as a neurotransmitter/neuromodulator in the CNS and autonomic nervous system [78,79,80,81]. It is a peptide highly expressed in the mammalian brain, which is involved in many brain functions, such as: motor activity, sleep, sensory, sedation, excitation, catatonia, body temperature, feeding, nociception, self-stimulation, seizure, learning, memory, and cognitive processes [12,82,83].

**Figure 1:**
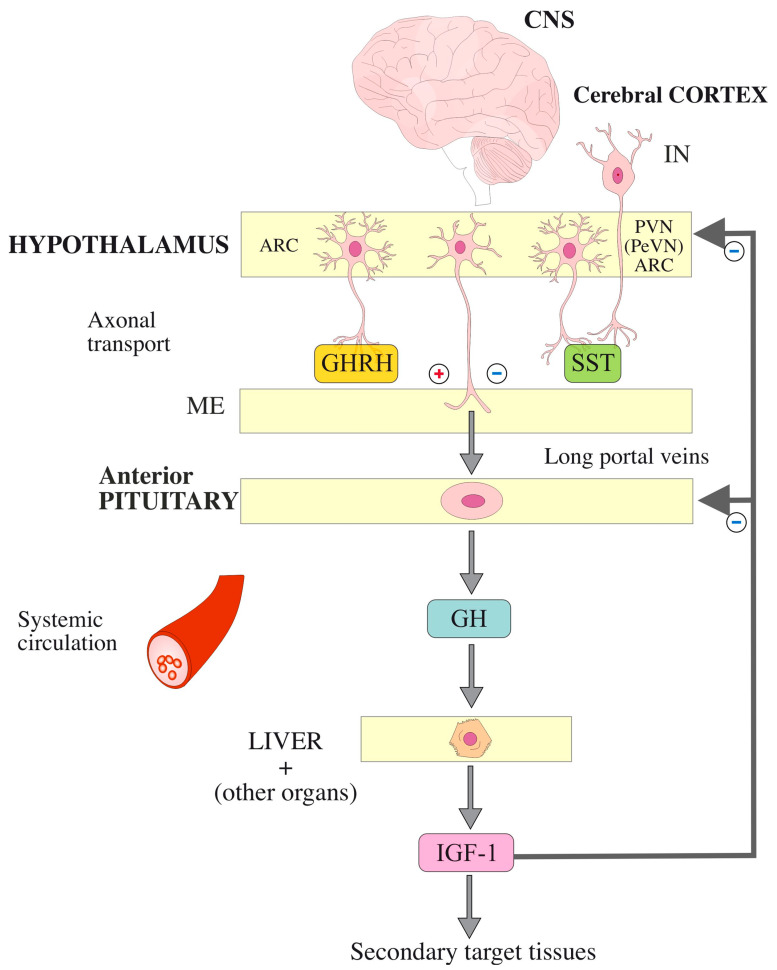
A schematic diagram of the hypothalamic–pituitary–somatotropic (HPS) axis. The hypothalamus releases GHRH (stimulating) and SST (inhibiting) to control pituitary GH secretion, which triggers IGF-1 release from the liver. SST-producing neurons are found mostly in the periventricular subnucleus (PeVN) of the paraventricular nucleus (PVN), but also in the arcuate nucleus (ARC). GHRH is produced mostly in the ARC. Their axons project mainly to the median eminence (ME), where they are released into the bloodstream and travel via the long portal veins to the anterior lobe (or *pars distalis*) of the pituitary gland. The anterior lobe of the pituitary contains somatotrophs that secrete GH in a pulsatile manner into the bloodstream. The primary target for GH is the liver, which stimulates the production and secretion of IGF-1. This peptide mediates the most growth-promoting effects on bone, muscle, and other tissues, while also providing negative feedback to the hypothalamus and pituitary gland to regulate the system [ARC—arcuate nucleus; CNS—central nervous system; GH—growth hormone; GHRH—GH-releasing hormone; IGF-1—insulin-like growth factor 1; IN—interneuron; ME—median eminence; PeVN—periventricular nucleus; PVN—paraventricular nucleus; SST—somatostatin].

In the CNS, SST is secreted not only in the hypothalamus, but also in other areas of the central and peripheral nervous systems [84,85]. Cell bodies of neurons are found mostly in the periventricular subnucleus (PeVN) within the paraventricular nucleus (PVN) of the anterior hypothalamus. SST is produced also in the arcuate nucleus (ARC) which is also a part of the periventricular zone of hypothalamus, and in parvocellular preautonomic neurons that reach the brainstem and spinal cord, including areas that control the sympathetic and parasympathetic nervous systems (reviewed in [86]). SST is produced also in the cerebral cortex, where it forms a population of inhibitory interneurons (SST-IN). These are further divided into Martinotti cells and non-Martinotti cells, which can be subdivided into long-range projecting IN and, among others, basket cells and double-bouquet cells [87] (Figure 1).

The mechanisms of GH inhibition in vertebrates by SST produced by PeVN are described extensively in numerous excellent reviews [43,76,88]. SST acts both centrally and peripherally, controlling the GH/IGF-1 axis [89]. In addition to inhibiting GH, SST also inhibits IGF-1 [90], the release of thyroid-stimulating hormone (TSH), prolactin (PRL), adrenocorticotropic hormone (ACTH), luteinizing hormone (LH), and almost all gastrointestinal and pancreatic hormones (e.g., gastrin, gastric inhibitory polypeptide (GIP), insulin, glucagon, cholecystokinin (CCK), and secretin) [6,7,91,92]. The short half-life of SST (2–4 min) and slight fluctuations in blood concentration may indicate that it acts mainly in a paracrine mode and in a neurocrine mode in close vicinity [10,78,93]. SST is a marker for representatives of the main class of inhibitory neurons in the mammalian cerebral cortex, i.e., γ-aminobutyric acid (GABA)ergic neurons (approx. 20% of the total neuronal network) [80,94]. It is also found in IN, which control the activity of neurons that stimulate the cerebral cortex. It precisely tunes the activity of neurons and participates in synaptic plasticity and memory formation (reviewed in [95]). The role of SST-IN, which can rapidly and reversibly silence excitatory synaptic connections also by regulating presynaptic release, and not only by rapid inhibition via GABA_A_, has been described [79]. SST-IN also modulate the network of the evolutionarily youngest and most developed part of the cerebral cortex (neocortex) via GABA_B_ receptors in a synapse-specific manner [81]. Activation of α-2 adrenergic receptors located in the brain stimulates GH secretion both by increasing GHRH secretion and by inhibiting SST secretion. In turn, activation of β-adrenergic receptors inhibits GH secretion by increasing SST secretion [96].

Although SST has been shown to influence the circadian rhythm of GH production in rodents [97], a progressive decline in the number of neurons expressing SST mRNA within subnuclei of the PeVN was observed in the brains of male mice aged 4 to 16 weeks (wks). Changes in SST expression in the PeVN and ARC therefore do not reflect the observed decline in pulsatile GH secretion in mice from puberty to early adulthood [98]. Similarly, in humans, it has been hypothesized that SST regulates the amount of GH released, but does not participate in controlling the rhythm of GH secretion [99,100]. The biological clock (circadian oscillator) in mammals is located in the suprachiasmatic nucleus (SCN) in the anterior hypothalamus, where the most significant neurons are those expressing arginine vasopressin (AVP) and SST [101]. In rats, SST-synthesizing neurons are GABAergic and constitute a distinct group of cells in the SCN, different from AVP or VIP/peptide histidine isoleucine amide (VIP/PHI) cell groups [102]. Recent studies in mouse models indicate that SST levels, daily rhythms, and the number of SST cells in the SCN are regulated by light, and that SST signaling acts to increase circadian rhythm resilience in a sex-differentiated manner. Light modulates the neurochemistry of central clock circuits, and long days increase SST expression. Furthermore, SST deficiency has been shown to increase circadian plasticity at the behavioral and cellular levels [103]. Changes in SST signaling at this level are associated with circadian and seasonal neuropsychiatric disorders in humans and rodents [12]. A mouse model has shown that low SST levels have a critical effect on mood-related phenotypes. Furthermore, impaired regulation of protein translation via eukaryotic initiation factor 2 (EIF2) may be a mechanism of SST neuron vulnerability [104].

### 3.2. GHRH/SST Axis in Aging

Brain aging differs from the aging of all other organs because neurons are highly differentiated postmitotic cells. The lifespan of most neurons in the postnatal period is equal to the lifespan of the brain (reviewed in [54]). As shown by Lu et al. two decades ago, the transcriptional profile of the frontal cortex of people aged 26 to 106 allows us to define a set of genes whose expression decreases after the age of 40. Among them is the *SST* gene (−2.9 fold changes) [105]. Study by Chen et al. in the frontal cortex of the senescence-accelerated mice/prone 8 (SAMP8) model during the aging process confirmed this result and showed −1.7 fold changes for SST. These results suggest that SST may play a key role in the process of brain degeneration in both mice and humans [106].

One of the most frequently studied brain structures affected by aging is the hippocampus, which is involved in various basic cognitive processes. The investigation is focused on the connection between the structural dynamics of IN (including SST-IN) during aging [107,108,109]. Dendritic spines are abundant in the SST-IN subpopulation as postsynaptic structures, particularly in *oriens-lacunosum moleculare* (O-LM) cells in the hippocampal CA1 region. Studies in mice have shown a significant decrease in the density of these spines in 9-month (Mo)-old animals compared to 3-Mo-old animals. With aging, there was a decrease in the expression of the N-methyl-d-aspartate receptor (NMDAR) subunit, i.e., glutamate ionotropic receptor NMDA type subunit 2B (GluN2B) in O-LM cells, but not the GluN1 subunit. The authors believe that changes in NMDARs affect not only pyramidal neurons, but also SST(+) cells, and consequently the inhibition that these IN exert on excitatory cells [109].

Analysis of tissue changes in NP expression (including SST) by nerve cells in different areas of the brain has revealed the varied impact of aging on this process [110,111,112,113,114,115,116].

#### 3.2.1. Animal Model Studies

In the hippocampus of rats, a varied, age-related decrease in the levels of selected interneuronal mRNAs of GABAergic molecular markers (including SST) was demonstrated, which was inversely correlated with the upregulation of the alpha 1 subunit of the GABA receptor. In older individuals, the most frequently altered mRNAs were SST and neuropeptide Y (NPY) (75% of animals), calretinin (CALR), VIP (50% of animals), and parvalbumin (25% of animals) [107].

In another study of tissue NP levels in rats aged between 4 and 18 Mo, increased NP levels were found in all four areas of the brain examined (frontal cortex, hippocampus, striatum, and hypothalamus). CRF and SST levels increased in the hippocampus, while NPY levels decreased. In older animals (18–26 Mo), significant changes in tissue protein levels were observed only in the hippocampus, but SST was not among them [114]. Martinoli et al. demonstrated sexual dimorphism in the expression of SST mRNA in the PeVN of the hypothalamus in adult rats (higher levels in males than in females). A gradual decline in SST mRNA was observed in middle-aged and aged rats of both sexes. No significant differences were reported among the three age groups (17, 20, and 27 Mo) considered. This would suggest that middle age and aging are critical periods for the control of *GH* and *SST* gene expression. However, the decline in GH mRNA levels observed during aging does not appear to be a consequence of increased SST activity [117]. In rats aged 3, 12, 20, and 30 Mo, a general decrease in SST and LHRH concentrations was observed with age in the ME [110]. Spik and Sonntag’s study in these animals showed a decrease in basal GH secretion both with age and after SST administration. Furthermore, in the presence of SST-14, GHRH-induced GH release was significantly attenuated in older rats compared to young or middle-aged rats. The authors believe that postreceptor changes in the pituitary response to SST contribute to the age-related decrease in GH secretion [112]. Similarly, in the brains and pituitary glands of male rats aged 3, 12, and 22 Mo, an age-related decline in SST and substance P content in the striatum and hypothalamus was demonstrated during the day. Differences in age-related levels of various NPs may vary further depending on the circadian rhythm [115]. Other authors observed a reduction in SST in older rats in the ME and an increase in its level in the anterior pituitary. The peak SST level in the ME occurred at the beginning of the activity phase (young rats) or at the end of the rest phase (older rats), and the SST peak in the pituitary gland occurred in the early stages of the rest period in older rats [118].

Studies by Ge et al. on in vitro hypothalamic explants in male and female rats showed that basal GHRH release did not differ significantly between both sexes, but at all ages, males released more GHRH in response to stimulation with both 28 and 56 mmol potassium/L than female rats. Neither basal nor potassium-stimulated GHRH release changed with age. In contrast, both basal and potassium-stimulated SST secretion increased significantly with age, but was the same in both sexes. GHRH content in the hypothalamus was significantly lower in 10-day-old rats compared to older rats, but remained constant after 30 days of age. SST levels gradually increased with age. The amount of both peptides in the hypothalamus was the same in both sexes. It was therefore concluded that male rats release more GHRH in vitro than females, which would reflect the increased amplitude of GH pulses observed in males in vivo. The progressive decline in GH secretion, previously described in the aging process, appears to be parallel to the progressive increase in SST release and levels observed in this in vitro system [119]. According to the one review [66], older rats exhibit significant changes in neurotransmitter and brain NP production in the hypothalamus and extrahypothalamic structures, which are most likely caused by defects in GHRH- and SST-producing neurosecretory neurons. GHRH synthesis is impaired in the hypothalamus of aging male rats, as indicated by a decrease in GHRH mRNA levels and GHRH-like immunoreactivity (ir). Although SST expression appears to decrease with age in the hypothalamus, the secretion and activity of this hormone are increased, resulting in a change in the relationship between *GHRH* and *SST* gene expression and their secretion. The findings of García-San Frutos et al. suggest that the age-related decline in GH levels may result from reduced GHRH function rather than increased SST activity. A decrease in the expression of the GH secretagogue receptor (*GHS-R*) gene in the hypothalamus may attenuate the effect of ghrelin on GH release. The age-related decline in GH/IGF-1 axis activity may be reversed by GHS treatment [120].

A study on two inbred mouse strains (C57BL and BALB/c) showed that central neurotransmitters and neuromodulators have different abilities to adapt to aging processes. This seems to depend on the genotype, CNS structure, and type of neurotransmitter. In adaptation to aging, striatal cholinergic activity and SST levels are more impaired in BALB/c mice than in C57BL mice. In contrast, in hippocampus cholinergic neurotransmission and SST levels did not differ with aging in either mouse strain [121].

Cardoso et al. found in a rat model that aging caused a decrease in the total number of NPY(+) and SST(+) neurons in the hippocampus, accompanied by a reduction in cholinergic branches. However, 24-Mo-old caloric-restricted animals retained a number of these peptide neurons and a density of cholinergic branches similar to that of 12-Mo-old control rats. This would suggest that the age-related reduction in the number of NP(+) neurons is not due to neuron loss and may be dependent on the cholinergic system. More importantly, caloric restriction has a beneficial effect on NPY(+) and SST(+) neurons and the cholinergic system, even when applied in old age [122].

In dogs of 15 different breeds, a total of 154 genes (77 genes with reduced expression and 77 genes with increased expression, respectively) were found to have a significant change in expression level with age. A significant overrepresentation of genes related to hormone transport (e.g., *SLCO1C1*, *EXOC3L1*, *NOS2*, *FKBP1B*, *PRKCE*, *SPP1*, *TNF*, *SLC44A4*, and *SLC16A2*) and hormone level regulation (*SLCO1C1*, *EXOC3L1*, *NOS2*, *FKBP1B*, *PRKCE*, *SPP1*, *LRAT*, *TNF*, *SLC44A4*, *PCSK5*, and *SLC16A2*, respectively) [55]. However, the *SST* gene was not among these genes.

A study by Nakamura et al. on the stalk-ME noted that mean GHRH levels during morning and evening in aged adult female rhesus monkeys (*Macaca mulatta*) were 3- to 4-fold lower than in young monkeys. Conversely, SST levels of aged and young monkeys were found to be twice as high in older animals than in young animals. Pulse analysis showed that SST amplitude and baseline levels were significantly higher in older monkeys than in young adults. No significant changes in the frequency of SST release pulses were observed [16].

Hypophysectomy caused a comparable decrease in GHRH in young (8 wks) and aged (6 Mo) rats, and the decrease in SST levels was greater in young than in aged rats. The SST/GHRH ratio increased in aged rats. After GH substitution, SST content did not change, while the SST/GHRH ratio returned to normal. Age-related differences in growth rate are accompanied by corresponding changes in the SST/GHRH ratio. Furthermore, SST and GHRH are regulated by short-loop feedback in both age groups, with an increased response in aged rats [123].

Morimoto et al. reported that neither the number of SST neurons nor the intensity of SST-ir was changed between young (3 Mo) and old (24 Mo) male rats in the hypothalamus. Intense SST immunoreactivity was observed in the outer layer of the ME in both groups of animals [111]. Other immunocytochemical (IHC) studies observed only a slight decrease in the number of SST-ir cells in the parietal and occipital cortex of the brain in a group of older rats [116]. IHC studies in female C57BL/6J mice showed an increase in the number of SST-ir neurons from 2 to 4 Mo, but a decrease after 4 Mo. It was observed that both GHRH-ir neurons and SST-ir neurons are less numerous in older female mice, but the ratio of SST/GFRH-ir neurons increases in older females. This suggests that the decline in the number and size of GH-ir cells in the pituitary gland with age may influence the increase in the ratio of SST-ir neurons to GHRH-ir neurons in the hypothalamus in female mice [124]. Another IHC study showed that in male mice, the number of GHRH-ir neurons also decreases significantly with age. Interestingly, the number of SST-ir neurons did not differ significantly between all age groups. The volume of the anterior pituitary lobe and the number of pituitary parenchymal cells decreased dramatically from 4 to 12 Mo. The percentage of GH-ir cells decreased significantly with age, as did the absolute number from 4 to 12 Mo and the size from 2 to 4 Mo and from 4 to 12 Mo. This suggests that the reduction in the number of GH-ir cells in male mice with age is modulated rather by a reduction in the number of GHRH-ir neurons than by SST-ir neurons [125].

In older rats (25 Mo), a significant reduction in pre-prosomatostatin mRNA levels was observed in the frontal cortex (−49%), parietal cortex (−80%), and striatum (−69%), but there were no significant differences in pre-prosomatostatin mRNA content in the hypothalamus. These results suggest that changes in *SST* gene expression may contribute to behavioral and cognitive impairments associated with aging [113]. Studies on hypothalamic and cortical tissue showed that in the cerebral cortex, the relative expression of the *SST* gene was similar in young (3–4 Mo), middle-aged (12–14 Mo), and old rats (22 Mo). However, *SST* gene expression in the hypothalamus decreased with age, and its level in older rats was approximately 50% of that observed in young animals. A single SST transcript of approximately 0.65 kb was detected in all age groups. Furthermore, it was demonstrated that the age-related decrease in SST mRNA expression in the hypothalamus is a consequence of reduced expression in specific hypothalamic nuclei, rather than a loss of SST(+) neurons [126]. Another study on SST mRNA showed a decrease in older rats, but interestingly, the amount of SST mRNA associated with polysomes increased in these animals. GH secretion dynamics decreased in young animals maintained on a moderately calorie-restricted diet, but after 26 months, the amplitude of the GH pulse increased and was indistinguishable from that of young animals fed ad libitum. Furthermore, animals subjected to moderate calorie restriction did not show a decrease in total SST mRNA or an increase in SST mRNA associated with polysomes, which is characteristic of 25-Mo-old animals fed ad libitum. These results suggest that altered regulation of SST mRNA at the translational level may be a contributing factor to the decrease in GH secretion observed in aging animals [127]. These studies suggest impairments in the mechanisms regulating translation in aging animals [127,128].

Age-related patterns of GH, IGF-1, and SST in serum, pituitary gland, and cerebral hemispheres were also studied in rats. Immunoreactivity and mRNA levels of both SST and IGF-1 were low in the cerebral hemispheres of aging rats, but returned to adult levels after GH administration. Since treatment did not cause changes in serum IGF-1 levels, these results demonstrate the stimulatory effect of peripherally administered GH on the regulation of *SST* and *IGF-1* genes in the CNS of aging rats [129]. A decrease in SST mRNA expression in some GABAergic IN and a decrease in the number of SST-ir cells in the hippocampus were demonstrated in older rats. Furthermore, in older animals, SST and IL-1β transcript expression were inversely correlated, and the decrease in the number of SST-ir cells was greater in the dentate gyrus than in the *cornu ammonis 1* (CA1) area. Finally, chronic intraperitoneal injections of lipopolysaccharide (LPS) in young animals mimicked age-related hippocampal inflammation as well as a decrease in SST mRNA expression. These results confirm that neuroinflammation is a potential factor involved in age-related degeneration of SST-GABAergic cells. The authors presented strong in vivo evidence supporting the thesis that age-related inflammation of the nervous system and degeneration of SST-GABAergic cells are two natural, related processes [130].

A summary of studies on SST expression/level in different brain regions in animal models in relation to age is presented in Table 1.

#### 3.2.2. Human Studies

No significant age-related changes in SST levels were found in the frontal cortex, caudate nucleus, putamen, medial globus pallidus, or substantia nigra of the human brain [131]. However, an analysis of the transcriptional profile of the frontal cortex revealed a significant decrease in *SST* gene activity after the age of 40 [105]. Recently, a comprehensive description of differences in various cortical cell types associated with human healthy aging was presented. The most significant changes were fewer SST-IN and VIP-IN, more astrocytes and other non-neuronal cells. The associations of cell types with age were clearly visible in aggregate and individual cell nuclei [132].

In young men (aged 20–24) and older men (aged 68–82), it has been shown that the primary cause of age-related decline in GH secretion in humans is the atrophy of somatotrophs (resulting from a reduction in endogenous GHRH secretion) [15]. No significant differences in serum SST levels were observed among different age groups in healthy female subjects; these levels were 25.2 ± 11 pg/mL for women under 45 years of age and 36.2 ± 10 pg/mL for women over 55 years of age [133]. Another study found significantly higher plasma concentrations of SST-like ir (SST-LI) in young, healthy individuals in the control group (aged 24–27) compared with older individuals (aged 47–73). A strong negative correlation was observed between SST-LI concentrations and age [134].

In summary, studies on age-related changes in SST expression in animal models (primarily male rats) have focused on various brain regions, resulting in a wide variety of findings. When considering the entire CNS, both a decrease in the number of SST(+) cells, SST production, and/or secretion (mRNA/protein) in the brains of aging rodents and the absence of statistically significant changes in young and old animals were observed. Interestingly, several research models have demonstrated an increase in SST secretion in the hypothalamus, pituitary gland, and/or hippocampus of rats [114,119,127]. It is worth noting that with age-related increases in SST in one brain region (e.g., hippocampus), a simultaneous decrease in SST expression was observed in another region (e.g., striatum) or no significant changes were found in yet another region (e.g., hypothalamus) [114]. When considering the hypothalamus alone, the results were also inconsistent. Some studies found no age-related differences in the number of SST-ir cells [111,113], while others reported a decrease [110,117,118,124,126]. In contrast, studies on the cerebral cortex of rodents mostly show a decrease in SST expression [106,113,116,129], or insignificant changes between groups of young and older animals [114]. Transcriptomic studies of various types of human cerebral cortex cells during physiological aging indicate a decrease in the number of SST-IN and VIP-IN, i.e., structures involved in non-hypothalamic SST production [132].

The discrepancies between findings showing a decrease in SST expression in the hypothalamus of rodents and an increase in SST secretion and activity in primates [16] may be explained, among others, by greater age-related damage to the GHRH system compared to the SST neurosecretory system in primates. Studies using ISH in rodents showing lower expression in aged animals may support the concept of age-related damage to GHRH(+) neurons as the cause of a substantial decrease in pulsatile GHRH release in primates as well, although this should be verified in both non-human primates and humans.

In humans of various age groups, no statistically significant changes in SST levels were observed within different CNS structures [131] or in serum SST levels in control groups [133,134], but reduced *SST* gene activity with age was described in the frontal cortex [105]. In investigating the causes of somatopause in men, it has been suggested that the primary cause is the atrophy of somatotrophs due to a reduction in endogenous GHRH secretion, rather than an increase in SST secretion with age [15]. ISH analysis of SST mRNA in the rat hypothalamus showed that the age-related decrease in *SST* gene expression results more from a decline in expression in specific hypothalamic nuclei than from the loss of SST-containing neurons [126].

A few authors [117,119] have investigated sex differences in age-related changes in SST expression in the hypothalamus/pituitary gland of rats from different strains. They did not observe significant sex differences in SST levels with age. It is difficult to interpret these findings based on such a small sample size. Physiological studies describe sex differences in SST content in the hypothalamic nuclei of rats (the higher number of SST-ir neurons in males compared to females), but this has not been studied across different age groups. Furthermore, estrogen (E_2_) and progesterone (P) may act synergistically on SST cells in the hypothalamic nuclei of females in physiology, suggesting that both sex steroids may contribute to the formation of the female-specific SST release pattern [135]. The aforementioned studies of SST levels in female monkeys [16] were also discussed in terms of the potential influence of female sex hormones on age-related changes in SST levels.

It is difficult to interpret the discrepancies in the results regarding SST expression in various brain regions (particularly the hypothalamus) in rodents and mammals. These differences may stem not only from different research models (in vivo/in vitro) or methodological errors. It is impossible to rule out species-specific factors (species and strains of rats and mice), sex, the influence of administering other neuropeptides and hormones, the experimental conditions themselves (hypoxia, inflammatory changes, neurodegeneration of neurons and interneurons), and the method of analyzing SST peptide/mRNA/serum, etc. For example, the SAMP8 mouse model [121] is characterized by various age-related neurodegenerative changes in the hippocampus at the transcriptional and translational levels of proteins (e.g., GFAP), mitochondrial dysfunction, lipofuscin precipitation, and increased oxidative stress, which may also influence changes in SST levels [106]. The organization of the mouse PVN is different, differing significantly from that of the PVN in male rats. These differences are particularly evident in the spatial relationships between the two main subgroups of neuroendocrine cells (large-cell and small-cell neurons) and the three populations of pre-autonomic cells with a descending course in the PVN [85]. Given the complexity of the issue, research on SST expression requires continuation and more standardized experimental conditions or the inclusion of healthy individuals of various ages.

## 4. Somatostatin in the Gastrointestinal Tract

### 4.1. SST in Physiology

**Figure 2:**
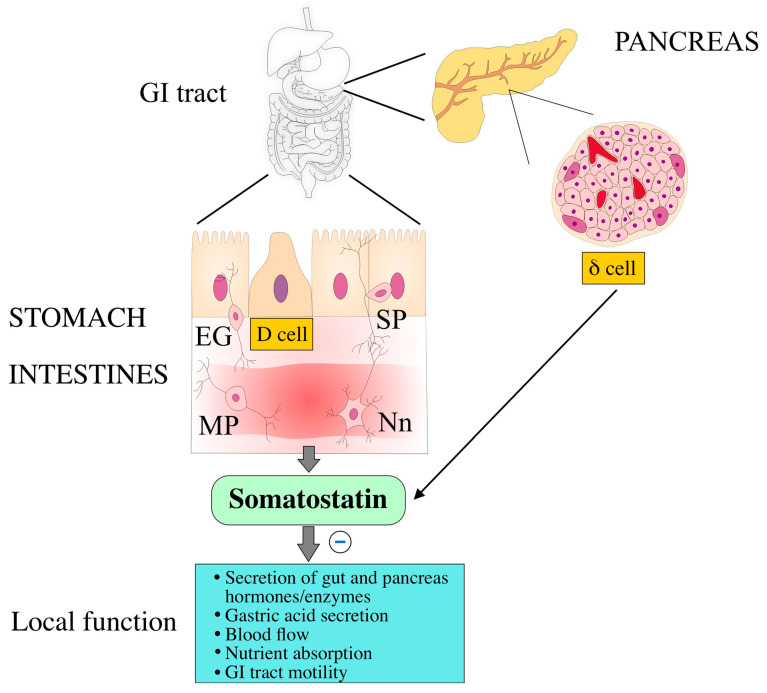
A simplified diagram showing the cellular sources and the functions of locally produced SST in the gastrointestinal (GI) tract [EG—enteric glia; MP—myenteric plexus; Nn—neuron(s); SP—submucosal plexus].

The presence of SST-ir cells has been demonstrated throughout the GI tract. The main SST-producing cells include mucosal enteroendocrine cells (EECs), termed δ (D) cells (stomach, colon, pancreas), and K cells (duodenum, jejunum), as well as neurons of the submucosal (SP) and myenteric plexuses (MP) [5,136,137,138] (Figure 2).

EECs, as a specialized population of intestinal epithelial cells, act as the “taste” cells of the mucosa, monitoring the contents of the intestinal lumen. They are an important link in the transmission of information from the intestinal epithelium to the CNS. They also have the potential to mediate the transfer of information from the brain to the intestines [139]. Studies on the distribution of EECs in the human intestine indicate that SST(+) cells are the dominant endocrine cells (alongside enterochromaffin and glycetin cells) also in the colon and rectum [140]. In the large intestine (LI) of cows and calves, the most frequent presence of argyrophilic cells and cells immunoreactive to many hormones (including SST) was also demonstrated in the rectum [141]. It should be noted that the majority of circulating SST is secreted by gastrointestinal D cells (~65%), ~30% by the CNS, and ~5% by pancreatic D (δ) cells [10].

The proportion of SST within the GI tract mainly concerns EECs (90%), with the remainder being enteric nervous system (ENS) structures (approximately 10%) [136,137,142,143,144,145,146]. The ENS is considered to be the second or intestinal “brain,” structurally and functionally resembling the CNS, as it has a high level of autonomy in the activity of intestinal neurons. It comprises over 500 million neurons and supporting glial cells, which are organized into separate layers or plexuses in the intestinal wall [146]. This system may also provide a window into the “first” brain. The ENS likely predates the CNS in evolutionary terms, and its complexity challenges that of its central counterpart, especially since the functional and chemical diversity of intestinal neurons closely resembles that of the CNS [147].

In the human stomach, less than 1% of myenteric neurons are morphologically multiaxonal type II neurons (putative afferent neurons). Most of them show reactivity to SST [148]. In the small intestine (SI), Dogiel type II neurons account for approx. 10% of the total myenteric neuron population and are characterized by co-expression of CALR, SST, and substance P [149,150]. In contrast, the submucosa of the SI and LI contains at least two distinct populations of neurons that differ in morphology and immunoreactivity for CALR and SST. A greater number of SST(+) neurons (predominantly unipolar) were found in the SI than in the LI. In the MP, CALR and SST coexist in Dogiel’s type II neurons [151]. According to other authors, submucosal SST-ir neurons in humans should be more accurately defined as “choline acetyltransferase (ChAT)(+)/SST(+)/substance P(±)”. Moreover, the authors suggest that one of the functions of human submucosal SST(+) neurons may be a primary afferent function [152]. Another study in humans observed very low concentrations of SST in the esophageal mucosa, which gradually increased in the gastric mucosa, mid-gastric and pyloric mucosa up to the proximal duodenum. Concentrations in the submucosa and muscle layer were generally low, except in the antrum and duodenum [153]. In the lower GI tract, SST was mainly found in the mucosa and submucosa of the sigmoid colon and showed low but constant concentrations in the muscularis externa [154].

The physiological role of SST in the GI tract in animals and humans is described in a number of reviews [4,5,6,155,156]. In brief, SST regulates intestinal motility and is a potent inhibitor of many GI tract functions, including secretion of digestive enzymes, nutrient absorption, blood flow in the intestines, hormone secretion (e.g., insulin, glucagon, and pancreatic polypeptide (PP) from pancreatic endocrine cells), and gastric acid production [3,5,92,157,158,159,160], as well as chloride secretion in the jejunum [161] and in the colon [162]. In the stomach, by binding to SST2 on G cells, it inhibits the release of gastrin, which leads to a decrease in gastric acid secretion and a slowing of gastric emptying [6].

SST also inhibits the release of cytokines from immune cells [163]. The anti-inflammatory effect of SST contributes to the integrity of the intestinal barrier. SST also reduces the secretion of amylase from the salivary glands, as well as hydrochloric acid, pepsinogen, and intrinsic factor from the GI tract mucosa [164,165].

Thus, the physiological effect of SST is to inhibit virtually all gastrointestinal functions as well as the exocrine and endocrine functions of the pancreas [156]. Gastric secretion of SST is stimulated by, among others, glucagon [166], gastrin and CCK [167]. Furthermore, SST and glucagon secretion are examples of reciprocal feedback, in which SST inhibits glucagon secretion at low and high glucose levels, and glucagon stimulates SST secretion via glucagon receptors and glucagon-like peptide 1 (GLP-1) receptors [166]. SST secretion is stimulated by glucose, fat, casein hydrolysate, and HCl administered into the stomach [168]. Other factors (e.g., insulin, glucagon, urocortin 3, and GABA) released by α or β cells of the pancreatic islets enhance the effect of glucose on SST secretion from δ cells, and SST acts locally as a paracrine or autocrine inhibitor of insulin. SST secretion is tightly regulated to ensure that digestive processes are neither overstimulated nor excessively inhibited. As the pH in the stomach returns to normal and nutrient absorption in the intestine proceeds, the stimulus for SST release weakens, allowing other digestive hormones to resume their activity. This negative feedback loop is essential for maintaining balanced and efficient digestion [10].

### 4.2. Age-Related Changes in SST(+) Cells

#### 4.2.1. Animal Model Studies

Age-related changes in the total number of SST(+) cell populations and their distribution in the rat stomach were described. It was shown that SST cells, invisible at birth, appeared in all rats on the seventh day after birth. Subsequently, their number increased more slowly but more regularly than G cells. During normal adulthood, the adjusted SST cell population was approx 130,000–200,000 cells. For the entire antrum, the G/SST cell population ratio decreased during the rat’s lifetime from 6.5 at 7 days of age to 1.5 in old age, with a stable value of 2.5 during adulthood [169].

The study of SST release from isolated perfused rat stomachs in three different age groups (4, 12, and 24 Mo) showed a decrease in basal SST concentration with age. Furthermore, bombesin-stimulated SST release decreased in 12- and 24-Mo-old rats. Carbachol inhibited SST release in each age group. These results indicate that the basal levels of gastrin and SST, which may influence pepsin secretion, were significantly lower in 24-Mo-old rats than in 12-Mo-old rats. In other words, the SST response decreased with age [170].

However, in studies on antral endocrine cells in a mouse model, a significant increase in the number of SST-ir cells was observed in mice aged 1, 12, and 24 Mo compared to young mice (3 Mo). Differences between various age groups were also observed in terms of the cell secretion index (CSI) of SST-ir and G-ir cells. The CSI of both SST-ir and serotonin(+) cells increased significantly in mice aged 1, 12, and 24 Mo compared to young mice. The observed changes would suggest that the large number of SST-ir cells in aging mice may contribute to the gastric delay observed in older individuals [171]. Endocrine cell types in the duodenum were also analyzed in the same four age groups of mice. The number of SST-ir cells was lower in both 12-Mo-old and 24-Mo-old mice compared to 3-Mo-old mice. In contrast, the CSI of GIP-ir and SST-ir cells was higher in 12-Mo-old mice compared to 3-Mo-old mice, and the CSI of SST-ir and serotonin-ir cells in 1-month-old mice was lower than in 3-Mo-old mice [172].

The plasma concentrations of several NP (including SST) were examined in extracts of various GI tract tissues in mice (stomach, duodenum, and colon) in four age groups (1, 3, 12, and 24 Mo). In the antrum and duodenum, lower SST concentrations were observed in 1-month, 12-Mo, and 24-Mo-old mice compared to 3-Mo-old mice. In contrast, lower SST concentrations were found in the colon of 1-month-old and 12-Mo-old mice compared to 3-Mo-old mice. It appears that the decrease in SST concentration in the colon of 12-Mo-old mice, without macroscopic signs of colon tumor, rather precedes tumor development than is secondary to the tumor. The decrease in SST concentration with age may therefore promote the development of CRC. This is also supported by the observation that in 24-Mo-old mice that did not develop CRC, SST concentration did not differ from that in 3-Mo-old animals [173].

An increase in the number of various colonic endocrine cells was observed in mice prior to puberty (1 month) as well as in older animals (12 and 24 Mo) vs. 3-Mo-old mice. This would suggest the role of enterohormones in the development of the GI tract, and in older age, indicate a role in compensating for increased receptor resistance and/or weakened response of effector organs [174]. In a study by Kvetnov et al. on BALB/c-nu mice aged 4, 21, and 34 Mo, an upward trend in the total number of gut neuroendocrine cells (NECs) with age was also demonstrated. Different types of gut NECs showed different behavior depending on age, and their course over time depends on location (stomach or duodenum). Although intestinal cells did not show significant physiological changes related to age (total number of epithelial cell nuclei, proliferating activity), modifications of specific biological parameters with age may suggest key compensatory mechanisms of regulatory processes during aging [175]. However, neither of these analyses included SST-ir cells. In contrast, the analysis of tissue expression of NPY, peptide YY, and SST, present in EECs in the colon mucosa in male rats of different ages, revealed no significant changes in the number of EECs expressing SST and chromogranin in younger rats compared to older rats [176].

Reviews on age-related changes in pancreatic islets mainly concern β cells [177,178,179], but only individual pancreatic D (δ) cells [180]. Age-related changes include β cell proliferation and apoptosis, which can affect their mass, resulting in changes in cell function at various stages. They can therefore affect, among other factors, electrical activity, Ca^2+^ signaling, and insulin secretion (reviewed in [179]).

The nutrient-induced release of insulin, glucagon, and SST in pancreas was studied in Fischer 344 rats aged 2, 10, 18, 24, and 30 Mo after overnight fasting. This pioneering study indicates that in rats, once adulthood is reached, there are only minor changes in SST secretion by δ cells. The secretory response of the pancreas remains constant throughout the life of these animals [181]. Another study analyzed the effect of donor age on exogenous inhibition of insulin secretion by SST stimulated by D-glyceraldehyde and β-D-glucose in the rat pancreas. It was shown that both doses of synthetic SST-14 used had a greater effect on glyceraldehyde- and glucose-stimulated insulin secretion in the pancreas of older animals (24–27 Mo) than in the pancreas of young animals (2–5 Mo). The authors conclude that increased sensitivity to the inhibitory effects of SST occurs during aging (as in pituitary tissues in vitro) [182]. Progressive histological abnormalities of the pancreas were observed with age, being more pronounced and severe in the senescent group of rats. Rats in this group had significantly increased volume density and B-cell density, as well as a greater number of pancreatic islet profiles compared to rats in the old group. A significant progressive increase in adipose tissue was also observed in older animals. No abnormal changes were detected in cell populations other than B cells in the different groups. The quantitative changes found in older animals suggest a possible compensatory response of the B cell population to limit the impact of diabetogenic factors that increase with age [183]. In studies by Cuesta et al. on SAMP8 mice, no age-related differences in SST expression in the pancreas were observed either [184]. Some reviews on this topic state that although the number of D (δ) cells in the pancreas does not change with age, there is an increase in secretory function that is more pronounced in older than in younger organisms. Due to their peripheral location, D cells are unlikely to inhibit B (β) and A (α) cells locally. In aging organisms, they may play a role in the development of involutional processes in the exocrine part of the pancreas. The process of secretory granule stasis in pancreatic A and B cells in older individuals does not appear to be the result of paracrine action of SST (reviewed in [180]).

#### 4.2.2. Human Studies

The relationship between SST concentrations and age in humans appears controversial, although higher SST levels have been observed in children between 1 and 2 years of age than in children over 10 years of age. It has been suggested that this may be related to the rate of growth and relative energy expenditure in early life [185]. However, RIA measurements of normal diurnal changes in VIP, SST, CCK, and PP concentrations in healthy volunteers showed an increase in basal and postprandial concentrations with age for PP only. Significantly higher PP concentrations in older individuals cause increased satiety, which may contribute to “age-related anorexia.” For both PP and SST, there was a significant diurnal rhythm in SST concentrations. SST levels were low in the morning and gradually increased throughout the day, reaching a peak at 7:00 p.m. However, increases after food intake were not significant. Average levels remained high at 11:00 p.m. No correlation was found between SST concentration and age. Unlike PP, SST was also not a major regulator of gastric cytoprotective trefoil protein/factor 2 (TFF2) levels in the gastric lumen [186].

IHC study involving the detection of SST-ir cells in the human rectum in three different age groups (20–29, 40–49, and 60–69 years) did not reveal any quantitative differences between the groups or between the sexes in terms of the number, types of EECs, and CSI. The cell nucleus volume was significantly larger in the 40–49 age group than in the other age groups. Thus, the observation from animal models of aging that age affects changes in the number of EECs in human LI was not confirmed [187]. IHC analysis in the pancreas of newborns (under 15 days old), infants (6 Mo) and adults with normal blood glucose levels showed a reduction in the percentage of SST cells (from approx. 30% in newborns to approx. 10% in adults) and an increase in insulin cells (50 to 70%). The percentage of glucagon cells remained stable (20%). In the posterior part of the pancreas, the percentage of PP cells was generally higher in adults than in newborns or infants. This study shows that both endocrine cell populations and the percentage of endocrine tissue in the pancreas undergo significant changes in early life [188]. A gradual and slight decrease in pancreatic islet cell volume density was observed, with a loss of both β cells and non-β cells. The mass of all these cells increases during puberty and gradually decreases after the age of 40 [24,189]. Other interesting findings suggest that a significant proportion of pancreatic islets develop a state of chronic low-grade inflammation with age, which may cause changes in functional plasticity. Changes in the expression of genes previously associated with T2DM and epigenetic changes important in explaining age-related dysregulation have been identified. The *SST* gene was not among those identified. Interestingly, this study shows that aging causes a relatively small number of transcriptional changes in the endocrine pancreas that are associated with the “inflammaging” phenotype and characteristics typical of T2DM [190]. It should be added that aging is also the most important risk factor for pancreatic cancer. Age-related pathological changes play a key role in pancreatic carcinogenesis through various mechanisms (reviewed in [24]).

### 4.3. Age-Related Changes in SST(+) Components of the ENS

The GI tract is innervated by intrinsic enteric neurons and extrinsic neural connections, including sympathetic and parasympathetic efferent fibers, as well as visceral afferent fibers, all of which undergo varying degrees of structural change with age (reviewed in [191]).

Numerous studies demonstrate the presence of SST in neurons and nerve fibers of the ENS in the SI and in different sections of the LI in various animal species under physiological and pathological conditions [142,145,192,193,194,195,196]. In the SI and similarly in the LI of the guinea pig, 5% of SST-ir neurons were observed in the MP and 17% in the SP [142]. Furthermore, cholinergic SST-IN in guinea pigs have been shown to have a unique cell body morphology and form networks of synaptic connections that run anally in the MP [194]. Other authors have visualized 18% of SST-ir neurons in the rat ileum and jejunum in the SP [193], and 1–6 neurons per ganglion in the colon MP of rats and mice [192]. SST-ir intrinsic neurons have long, granular axons in the enteric MP, as well as outside the plexus, between the longitudinal and circular muscle layers [192]. In the porcine descending colon, processes such as chemically driven inflammation (CDI), proliferative enteropathy (PE), and nerve damage (axotomy) resulted in a decrease in the number of SST-LI nerve fibers in the mucosa. However, in the circular muscle layer, CDI and axotomy increased the number of SST-LI nerve fibers, in contrast to PE, which reduced the number of such fibers. These results suggest that SST-LI neural structures in the ENS may be involved in various pathological conditions within the porcine descending colon, and their functions likely depend on the type of pathological factor [196]. According to the results of studies conducted on the ascending colon in the colitis model caused by *Bacteroides fragilis* [195] and IHC analysis of different NP in both SP (inner and outer) as well as in neuronal fibers in the colon of piglets, the number of observed SST-LI neurons was relatively low in both types of SP [145].

Studies examining changes in the ENS during aging suggest that enteric neurons are more susceptible to age-related degeneration and cell death than neurons elsewhere in the nervous system. Furthermore, there is considerable variation in the extent and time course of age-related loss of enteric neurons reported across studies. However, the aging gut appears to maintain its function remarkably well in animals exhibiting significant neuronal loss, suggesting that the ENS has significant functional reserve (reviewed in [197]). The mechanisms of aging of ENS components, age-related morphological changes and the metabolic effects of these changes are presented in the latest excellent reviews [198,199,200]. Structural and cellular changes include the percentage of intraganglionic neurons and the density of enteric neurons packed into ganglia (reduction), ganglia area (increase), percentage of ganglia with empty spaces (increase), postganglionic neuron population (increase), and regional density of S100(+) enteric ganglia (decrease). Dystrophic features of swollen axons and nerve endings are observed [198,199].

#### 4.3.1. Animal Model Studies

SST release is controlled by the vagus nerve [201] and the α-adrenergic system [96]. NPs such as substance P and SST have been shown to be synthesized in the cell bodies of the sensory ganglia of the vagus and sciatic nerves and transported bidirectionally to the CNS and sensory innervation sites. In a rat model (4, 12, and 25 Mo), SST levels in the sensory vagus nerve were shown to be unchanged with age, but their transport increased in older rats. However, in the sciatic nerve, SST content decreased by more than 30% in older rats, and transported SST decreased. Somatostatinergic neurons of the dorsal root ganglion appear to be particularly susceptible to aging in Fischer rats [202]. Ultrastructural studies of the SI in rats of different ages revealed SST-ir neuronal bodies primarily in substance P, and SST-ir nerve fibers primarily in MP and in the mucosa around the crypt bases. In aged animals, a reduced overall number and degeneration of nerve fibers immunoreactive to selected NP (including SST) were demonstrated in the SI compared to young and aged animals. This change could result in changes in epithelial transport processes associated with aging, as well as impaired intestinal motility in the GI tract [203]. However, there are also observations that do not confirm a significant age-related reduction in the number of neurons with a positive IHC staining for neuronal markers (including SST), except for approx. 15% reduction in the number of neurons with NADPH-diaphorase activity in old rats. Moreover, the study failed to observe extensive cell loss in the MP of old rats, both in the general populations and in any of the major functional groups of neurons [204].

A relatively recent study by Emanuilov et al. showed a significant increase in the percentage of SST-ir neurons in MP in SI and LI from early postnatal development to 20 days of age, remaining stable until 60 days of age. Subsequently, the percentage of SST-ir cells decreased in MP in both these locations in 2-year-old animals. Similarly, in SP, the percentage of SST-ir neurons increased significantly from birth to 20 days of age (SI) or 30 days of age (LI), after which it decreased in 2-year-old animals in SP located in SI and LI. A reduced percentage of SST/ChAT neurons was also observed in aged rats in both types of neural plexuses in SI and LI. In contrast, in young rats, only a few SST-ir neurons colocalized with nitric oxide synthase (nNOS), and this percentage increased significantly in 2-year-old rats. Colocalization of SST with glial fibrillary acidic protein (GFAP) was not observed in any of the animals studied. These studies confirm that SST expression in enteric neurons increases in young rats and decreases with aging. This process is accompanied by changes in SST colocalization, primarily with ChAT and nNOS [205].

Summary of studies on SST expression/level in different sections of the GI tract in animal models in relation to age is presented in Table 2.

#### 4.3.2. Human Studies

SST plays a role in the conduction of sensory and pain stimuli and the regulation of local intestinal motility, modulation of the release of other neuronal factors, and regulation of blood flow in intestinal vessels [136,206]. Furthermore, SST directly inhibits intestinal inflammatory responses by disrupting communication between mucosal mast cells and neurons. It also exerts profound analgesic effects by modulating extrinsic afferent nerve fibers [4].

In surgical patients of various ages, the effects of aging on the levels of neuropeptides (including SST) and on the innervation of inhibitory neurons within the circular muscle of the normal descending colon were examined, revealing no differences in their levels between patients aged <70 years and those aged ≥70 years. Only the amplitude of inhibitory nerve potentials decreased with the patient’s age, with no changes in resting membrane potentials. The decrease in amplitude in women preceded that in men. This was attributed, among others, to a reduction in the density of inhibitory nerves, a decrease in the release of inhibitory neurotransmitters, or changes in the interaction of the inhibitory neurotransmitter with the membrane of smooth muscle cells [207]. The presence of SST(+) nerves was detected also in human gastric antrum, duodenum, jejunum, ileum, ascending and sigmoid colon, and rectum. Numerous SST(+) perikarya and small varicose fibres were observed in the intestine. SST was detected in mucosal fibers which formed a subepithelial plexuses and in the *lamina propria* of the mucosa. Positive perikarya and nerve endings were also observed in the submucosal ganglia. A small number of nerve fibers were associated with submucosal blood vessels. In MP, mainly SST(+) nerve endings were detected, not perikarya, but their overall number was lower than in SP [143]. However, this study does not describe changes associated with age. It is worth remembering, however, that human pathologies affecting enteric neurons may reflect central changes in neurodegenerative disorders [208] or in cancers, including CRC [209]. On the other hand, aging phenotypes and pathophysiological mechanisms of ENS aging highlight the role of enteric neurons in age-related CNS diseases such as Alzheimer’s disease (AD) or Parkinson’s disease (PD) [198].

In summary, studies on age-related SST expression have been conducted in various segments of the GI tract (stomach, duodenum, colon) and the pancreas, primarily using animal models (rats and mice) and predominantly employing IHC techniques. Varied results were observed (decrease/increase/insignificant changes), with a predominance of age-related decreases in SST tissue expression. These studies were conducted in the entire organ, in EECs, and in components of the ENS. In humans, there is a lack of research on SST expression/level in the aforementioned segments of the GI tract related to age.

With regard to the pancreas in animal models (rats), histoarchitectural damage, impaired pancreatic islet function, and increased sensitivity to the inhibitory effects of SST were observed during aging. As the animals aged, a loss of both β-cells and non-β-cells was observed, though this was not always accompanied by a significant decrease in SST release from δ-cells [182]. Similarly, in humans, a reduction in the proportion of SST(+) cells in the pancreas was observed [188]. Furthermore, a state of chronic low-grade inflammation developed in a significant portion of the pancreatic islets, which could cause changes in functional plasticity with age. Genetic and epigenetic changes associated with the development of T2DM and important for explaining age-related regulatory disorders have been identified, but they did not involve the *SST* gene [190].

Variations in the results of studies regarding changes in SST levels in the GI tract structures of animals and humans with age are most likely due to differences in methodology (IHC, RIA, light/electron microscopy), species differences and individual characteristics of animals, e.g., the relatively short lifespan of rodents, varying metabolic rates, and different GI tract physiology (different neurohormonal regulation, e.g., the absence of motilin in rodents, differences in the expression and function of receptors for neurohormones such as serotonin, melatonin, or NPY, etc.) [210].

## 5. Somatostatin and Selected Age-Related Diseases

### 5.1. Alzheimer’s Disease and Senile Dementia of the Alzheimer Type

SST has been shown to play a significant role as a modulator of cognitive functions, learning, and memory. SST(+) neurons play a significant role in brain function by regulating hippocampal activity and memory formation (reviewed in [211]). A decrease in SST levels is observed in the aging brain and in various neurological disorders in older adults, namely AD and dementia [94,113,129,211]. Recent spatiotemporal comparisons of cell type proportions, gene expression, and cell–cell communication from two individuals with Braak III and Thal 4 AD showed differences in the vulnerability of SST and SST-chondrolectin inhibitory neurons and the expression of endosomal and lysosomal trafficking and metallothionein genes within the β-amyloid (Aβ) plaque microenvironment [212].

Senile dementia of the Alzheimer’s type (SDAT) is characterized by degenerative changes primarily affecting SST(+) neurons. Morphological changes in the neuronal population in the cortex and hypothalamus in SDAT differed from those observed in the brains of older individuals without CNS disease. SDAT is thought to be associated with anatomical changes and biochemical imbalances in multiple NP and neurotransmitter systems [213]. Nakamura et al. demonstrated that the density of SST-ir cells in the neocortex was not reduced in SDAT cases compared to healthy individuals. However, many SST fibers with abnormal morphology were observed, often present within senile plaques. SST(+) cells in healthy older individuals were distributed from layer II to the subcortical white matter. Morphologically, multipolar cells predominated. Therefore, the authors suggest that the primary degenerative process in SDAT may begin in SST(+) fiber terminals of the neuronal system in the neocortex [214].

Neuropathological phenomena occurring in AD include aggregation of Aβ plaques and tau-containing neurofibrillary tangles, age-related structural and functional deterioration, neuronal loss, dendritic reduction, axonal degeneration, and synapse loss. These pathological features are observed in the medial prefrontal cortex (reviewed in [215,216]). In a transgenic mouse model of AD, the number of SST(+) neurons decreased with aging, and this decrease was more dramatic compared to controls. Aβ aggregation occurred with age in the granule cell layer, which may be related to the specific involvement of SST-expressing cells. These results support the idea that SST(+) neurons are early and preferentially involved in Aβ pathology in AD [217]. Regarding the mechanisms of action of SST-IN in AD, it has been hypothesized that age-dependent reduction in SST causes Aβ accumulation in the brain by inhibiting the action of neprilysin [215]. Neprilysin is the main enzyme that degrades Aβ, and its activity is enhanced by SST [218,219,220]. However, an in vivo study in mice demonstrated that SST had no effect on transcription levels, protein levels, or neprilysin activity in the whole brain, which cannot be explained by compensatory upregulation of the SST paralog, CST, observed in 15-Mo-old SST-deficient mice. This deficiency led to a subtle but significant increase in Aβ plaque density in the cerebral cortex. Subsequent analyses demonstrated that SST interferes with the early stages of Aβ formation. No effect of SST on steady-state tau levels or its phosphorylation was observed. This supports data indicating that SST influences Aβ plaque formation by directly interfering with Aβ aggregation [221]. SST has been experimentally shown to disrupt Aβ fibrillization and promote the formation of 50–60 kDa Aβ complexes. The distribution of SST and Aβ overlaps in the brain, and SST has been linked to AD based on several additional observations. It is noteworthy that SST is one of several NPs that acquire amyloid properties prior to their synaptic release (reviewed in [219]). The main characteristics of progressive AD include altered SST levels and the colocalization of SST-IN with Aβ plaques, leading to cell death. Based on available research, an interesting molecular model of AD pathogenesis involving SST has been constructed (the somatostatinergic model of AD (reviewed in [222,223]). Both hyperactive and hypoactive SST-IN struggle to control hyperactivity in medial areas in early stages of AD, leading to axonal Aβ production through excessive presynaptic GABA_B_ inhibition, downregulation of the GABA_B1a_/amyloid precursor protein (APP) complex, and internalization. Simultaneously, excessive SST-14 release accumulates near SST-IN as amyloid, which binds to Aβ, forming toxic mixed oligomers. This leads to differential death of SST-INs through excitotoxicity, further disinhibition, SST deficits, increased Aβ release, fibrillation, and plaque formation. On the other hand, chronic stimulation of postsynaptic SST2/4 receptors on glutamatergic neurons by hyperactive SST-IN causes intense activity of p38 mitogen-activated protein kinase (MAPK), which leads to somatodendritic p-tau staining and apoptosis/neurodegeneration [222].

Numerous studies have implicated reduced SST levels in the pathogenesis of both AD and SDAT in humans [215,219,224,225,226,227,228]. RIA studies have shown reduced SST immunoreactivity in the cerebral cortex in tissues of AD/SDAT patients [224]. The decrease in ChAT activity observed in the temporal cortex of SDAT survivors was accompanied by a significant reduction in SST-ir cells (47%) [225]. In contrast, IHC studies have demonstrated the presence of SST-ir cells in neuritic plaques in individuals with AD. Analysis of the colocalization and distribution patterns of plaques and SST suggests that AD neuropathology may be primarily associated with the loss of selected cortical neurons that are targets of the involved neurotransmitter systems. Plaque formation, in turn, is thought to result from degeneration of presynaptic and postsynaptic axons of large projection neurons in layers III and V [229]. Furthermore, fewer SST-INs per mm^2^ were identified in the temporal cortex of AD patients compared with control subjects. These results support a regional neuroanatomical influence on selected classes of INs in AD and suggest that impairments in interneuron circuitry may contribute to neuronal dysfunction and cognitive decline in AD [230]. Using all-atom molecular dynamics simulations of model mixtures of Aβ42 or Aβ40 peptides with SST-14, it was shown that the presence of SST-14 impedes aggregation in the Aβ42–SST-14 system despite its high hydrophobicity, inducing a stronger “sticky surface” effect in aggregates at the onset of Aβ42–SST-14 oligomerization [231].

A very interesting recent study has identified diverse cell populations associated with AD, including a subtype of neurons with SST inhibitory activity and oligodendroglial neurons. Furthermore, a network of multicellular populations was identified, each composed of coordinated subpopulations of neurons, glia, and endothelial cells. Two of these populations are altered in AD [232]. A very interesting single-cell transcriptomic atlas of the aging human prefrontal cortex was also created, encompassing 2.3 million brain cells from individuals with varying degrees of AD pathology and cognitive impairment. The authors identified, among others, selectively sensitive subtypes of SST inhibitory neurons, which are reduced in AD. They discovered two distinct groups of inhibitory neurons that were more numerous in individuals with preserved high cognitive function in old age, as well as a link between inhibitory neurons and resistance to AD pathology. Interestingly, gender differences in response to AD have been demonstrated [228].

### 5.2. Parkinson’s Disease

Aging is also the greatest risk factor for the development of PD. A significant feature of PD is reduced SST levels in the brain [226,233] and in cerebrospinal fluid (CSF) [234]. It has also been shown that in the early stages of untreated parkinsonism, SST levels in the CSF may increase, likely due to neurodegenerative depletion of SST from striatal or cortical neurons [235]. Significantly reduced SST levels have been observed in the frontal cortex of PD patients with mild or severe dementia compared to controls and PD patients without dementia. Significant reductions in SST levels have also been noted in the hippocampus and entorhinal cortex (EntCx) of patients with severe dementia [233]. In this disease, substance P expression is decreased in the basal ganglia, while SST is decreased in the neocortex. This suggests that substance P imbalances are associated with movement disorders, while SST deficiencies are associated with cognitive impairment [226].

The main characteristics of PD are extrapyramidal motor signs [236,237,238]. Experimental studies in animal models (rats) indicate that both these factors, i.e., aging and SST deficiency in the brain, can lead to catalepsy [236,237]. A pioneering study demonstrated that the *locus coeruleus* (in the dorsal pons) is a brain center capable of inducing catalepsy and may be a potential target for therapeutic manipulation of extrapyramidal dysfunctions [237]. Subsequent studies demonstrated that the development of catalepsy in aged rats appeared to be associated with hypoactivation of the lateral EntCx (LEntCx). It is likely that there is a mechanistic link between LEntCx hypoactivation and increased susceptibility to catalepsy in aged rats [238].

SST belongs to the group of NPs that plays a significant role in neuronal protection in both in vivo and in vitro models of PD (reviewed in [239]). The neuroprotective mechanisms of SST in PD were investigated by the group of Bai et al. [240]. The authors demonstrated an inhibitory effect of SST on LPS-induced microglial activity and the production of reactive oxygen species (ROS). Furthermore, SST inhibited or attenuated the production of proinflammatory cytokines: tumor necrosis factor-α (TNF-α), interleukin 1β (IL-1β), prostaglandin E2 (PGE2), and the expression of inducible NO synthase (iNOS), cyclooxygenase-2 (COX-2), and nuclear factor κB (NF-κB) p-p65, induced by LPS. These results indicate that the observed neuroprotective effect of SST was associated with the inhibition of microglia activation and the NF-κB pathway, which resulted in reduced neuroinflammation and oxidative stress [240].

SST mRNA levels in GABAergic-IN derived from induced pluripotent SCs (iPSCs) from PD patients with *PARK2* mutations have also been shown to decrease with progressive neuronal maturation. The number of GABAergic SST-IN was also observed to be significantly reduced in GABAergic-IN derived from iPSCs from patients with *PARK2* mutations. This suggests that reduced SST expression in GABAergic-IN in PD may, at least in part, lead to the complex symptoms of this disease [241]. A recent excellent review [242] critically analyzed the role of NPs (including SST) as biomarkers for early PD diagnosis.

AD and PD are also accompanied by neuronal loss in the colonic MP and SP of the ENS [208]. However, studies of NP expression (including SST) in PD have shown that only the VIP neuronal system is crucial in the disease process [243]. Furthermore, increased serum SST concentrations were observed in patients with early PD before and after treatment with antiparkinsonian drugs in patients who experienced nausea or vomiting compared to healthy individuals [244]. Further studies on the role of this NP in PD are necessary to clarify the regenerative capacity of SST in PD [239].

Summary of studies on SST expression/level in different models of selected age-related diseases of the CNS (AD, SDAT, and PD) is presented in Table 3.

### 5.3. Obesity

Aging is associated with increased visceral obesity (VO), which is a major risk factor for insulin resistance and metabolic syndrome (MetS). Obesity in older adults is a serious problem, and as recent reviews show, age-related changes in fat distribution and metabolism are key factors in a vicious cycle that can accelerate the aging process and the onset of age-related diseases [64,245,246,247].

Numerous studies have shown that SST inhibits nutrient absorption, suggesting that it may act as a physiological regulator of nutrient homeostasis by modulating the rate of intestinal absorption [156]. Changes in tissue SST content and plasma concentration have been observed in obesity, suggesting that SST deficiency may be a pathogenic factor. The observed SST changes appear to be secondary to changes in other functions. However, hyposomatostatinemia may facilitate nutrient absorption [155,156].

As suggested by other authors, VO seems to be the main factor inhibiting GH production, as the relationships between GH secretion and age, testosterone levels, or sleep are weakened or disappear with an increase in body weight. This is confirmed by data obtained using pulsatile GHRH administration or pharmacological methods that reduce SST secretion. The study results point to combined abnormalities in GHRH release and excess SST as the most likely pathophysiology of hyposomatotropism associated with obesity [248]. The accumulation of visceral fat may inhibit GH secretion through a variety of mechanisms, including increased levels of insulin and free fatty acids, decreased levels of ghrelin, and increased SST tone. In turn, relative GH deficiency may exacerbate VO due to impaired hormone-sensitive lipolysis [246]. Interesting review by Stengel et al. indicates that activation of the SST2 signaling in the brain via intraventricular administration of stable SST agonists strongly stimulates food intake and, independently of this, drinking behavior in rodents. The appetite-stimulating response involves downstream signaling pathways of orexin-1, NPY1, and the μ-receptor, whereas the thirst-stimulating effect is mediated by activation of the angiotensin 1 receptor in the brain. Activation of SST2 in the brain may also counteract the anorexic response to visceral stressors (reviewed in [249]).

The study of Zhu et al. showed that activation of basal forebrain (BF) SST neurons specifically induced high-calorie food intake without affecting normal food intake. This may be related to stress-induced overeating of palatable food, which contributes to obesity problems. Comprehensive whole-brain mapping of inputs and outputs revealed that BF SST neurons form bidirectional connections with several brain regions relevant to feeding and emotion regulation [250]. Using single-cell RNA sequencing, clusters of glucose-dependent insulinotropic polypeptide receptor (*Gipr*) cells were identified in human and mouse hypothalamus that exhibited transcriptomic features characteristic of SST(+) neurons, vascular cells, and glial cells. It was shown that activation of *Gipr* neurons using local adeno-associated virus (AAV) expressing Cre-dependent G_q_-DREADDs reduced food intake [251].

The role of SST in controlling food intake, energy storage, and expenditure is widely debated. A comprehensive review of Kumar and Singh describes the role of SST as a bridge between central and peripheral tissues, playing an important role in obesity behaviors related to food intake and energy expenditure. SST, together with ghrelin, is known to have an appetite-stimulating effect in humans, increasing food intake. SST alone exerts a dual effect (appetite stimulant and appetite suppressant). The mechanisms by which SST regulates satiety involve delayed gastric emptying and intestinal motility, which directly affect brain regions such as the hypothalamus, hippocampus, cerebral cortex, and brainstem (reviewed in [252]).

There are some studies examining the relationship between age-related changes, obesity, SST levels, and sex differences [20,43,64,253]. A randomized controlled trial conducted on 21 healthy men (18 Caucasians, three Asians) demonstrated the complex influence of sex hormones, GHRH, SST, IGF-binding protein 1 (IGFBP-1), and IGF-1 on ghrelin dose-responsiveness. This suggests a multifaceted modulation of the action of hormone-releasing substances. An environment with a high testosterone/estradiol (T/E2) ratio significantly attenuated the inhibitory effect of SST on ghrelin efficacy. GHRH selectively enhanced the dose-dependent potency of ghrelin. Age, IGF-1, and IGFBP-1 were found to be strong predictors of ghrelin potency or ghrelin-GHRH synergy in older men [20]. A recent study examined sex differences in the effects of nutritional factors on key components of the somatotrophic axis. It was demonstrated that early overfeeding had long-lasting effects on key components of this axis, which differed by sex, with a significant suppression of pituitary gene expression for GHRH-R and SST5 in men, but an increase in the expression of GHRH receptor (GHRH-R), SST2, SST5, GHS-R, and ghrelin in females. Importantly, early overfeeding sensitized the GH axis to the deleterious effects of a high-fat diet (HFD), causing significant inhibition of GH expression in the pituitary in both sexes and a reduction in blood GH levels in females. However, despite similar metabolic disturbances, quite distinct changes in key somatotropic regulators/mediators were observed in men and women [253].

### 5.4. Diabetes Mellitus

Risk factors for developing diabetes include both obesity and advanced age. Age itself is an etiologic factor in non-insulin-dependent diabetes mellitus (“age-related DM” or T2DM). Importantly, however, in older adults, β-cell dysfunction and deficiency play a greater role in the pathophysiology of diabetes than in younger adults, and muscle insulin resistance increases even in non-obese individuals [254,255]. Cellular senescence may result in a decline in β-cell function and represent a new and promising approach to restoring insulin secretion [256]. Others consider that cellular senescence is a programmed phenomenon that facilitates mammalian embryonic development and postnatal β-cell functional maturation. Therefore, it has been suggested that cellular senescence is involved in β-cell regeneration, insulin secretion, and the development of diabetes, although the exact mechanisms are still being investigated [178]. β-cells from mature mice and humans have been shown to secrete more insulin than young β-cells in response to high glucose concentrations, potentially counteracting age-related peripheral insulin resistance [257]. This change is likely orchestrated by cellular senescence induced by the cyclin kinase inhibitor p16^Ink4A^ and subsequent remodeling of chromatin structure and DNA methylation, which enhances the expression of genes controlling β-cell function. Activation of the cellular senescence program is thought to induce lifelong functional maturation of β-cells, driven by β-cell hypertrophy, increased glucose uptake, and more efficient mitochondrial metabolism, in parallel with the locking of these cells into a non-replicative state. Other age-related mechanisms act to increase basal insulin secretion even under low glucose conditions. This leads to a general reduction in the amplitude of insulin secretion between low and high glucose concentrations in old age, which may result in impaired metabolic control (reviewed in [177]).

It was hypothesized quite early that age-related insulin resistance is caused by a decline in mitochondrial function [254]. Recent studies suggest that β-cell aging is caused by endoplasmic reticulum (ER) stress, oxidative stress, and mitochondrial stress, contributing to β-cell dysfunction and impaired glucose homeostasis (reviewed in [258]). Another review confirms that β-cell function declines with aging in humans, independent of peripheral insulin resistance, BMI, and waist circumference. As observed in rodents, basal insulin secretion increases with age, with no change or an increase in stimulated insulin secretion. The accumulation of senescent β-cells may explain some of these functional changes. Transcriptional analysis of senescent β-cells revealed parallel downregulation of several steps in the pathway linking glucose stimulation and insulin secretion. Moreover, specific elimination of senescent cells (senolysis) improved the remaining β-cell function, gene expression profile and blood glucose levels [256].

Investigating the molecular mechanisms of age-related changes in pancreas, first in a rat model and then in a mouse model, confirmed a progressive age-dependent decline in insulin mRNA levels by 50% and 40%, respectively. However, SST levels did not change with age in both animals. The authors concluded that pancreatic endocrine activity declines with age, but these changes primarily affect β cells [259,260]. In turn, in Zucker diabetic and obese rats (ZDF) (an animal model of T2DM), the distribution of pancreatic hormones was shown to change to varying degrees and during the normal aging process. Interestingly, the percentage of SST(+) cells remained unchanged in young and older ZDF rats compared to age-matched controls (Zucker lean group) [261]. Similarly, previous studies have shown negligible changes in the number of SST-producing endocrine cells in aged animals. Even in 32-week-old ZDF rats, changes affected only β-cells and not other endocrine cells of the pancreatic islets. In control rats, aging induced only minor changes. During the long-term development of diabetes in both male and female ZDF rats, histopathological changes in the pancreatic islets ultimately led to islet disintegration [262].

The abnormal hormone secretion in T2DM cannot be attributed to significant changes in their pancreatic reserves. Lower SST levels were found in subjects with T2DM than in non-diabetic (ND) individuals (0.027 vs. 0.038 mg), but the SST/insulin and SST/glucagon ratios did not differ. Age tends to cause a slight decrease in insulin, glucagon, and SST contents of the pancreas in the entire group of ND subjects, but the correlations did not reach statistical significance. In contrast, aging was associated with a decrease in pancreatic content of the three hormones in T2DM [263].

Recent studies suggest that increased glucagon secretion and decreased SST secretion by the pancreas contribute to hyperglycemia in patients with T2DM. Studies by Kothegala et al. indicate a reduced number of δ cells and lower SST secretion in pancreatic islets in patients with diabetes. The δ-cell numbers were surprisingly reduced in human islets from donors with T2DM, and the reduction mainly affected the peripheral distribution of δ-cells in human pancreatic islets. This confirms that the decreased SST mRNA expression contributes to fewer δ-cells in human T2DM islets [264]. These results are consistent with transcriptomic data [265]. The study by Yang et al. using real-time detection of SST release from individual pancreatic islets, although supporting the above observations of reduced δ-cell numbers in pancreatic islets from donors with T2DM, showed that T2DM is associated with increased SST secretion, which influences the paracrine regulation of insulin and glucagon secretion [266].

The potential link between reduced SST expression and diabetic retinopathy is debated [267]. Significantly lower levels of SST mRNA and SST-28-ir have been demonstrated in both the retinal pigment epithelium and neural retina from diabetic donors compared with ND donors. Furthermore, increased GFAP and higher rates of apoptosis were observed in diabetic retinas compared with ND retinas. These changes were particularly pronounced in patients with higher SST deficiency [268].

### 5.5. Colorectal Cancer

CRC is a cancer that affects middle-aged and older adults, reaching a peak incidence between the ages of 50 and 70 years [25,26,173]. Higher incidence and mortality rates from CRC are observed in men compared to women [26,247]. Differences between men and women with CRC also extend to the epidemiology, clinical course, certain genetic and epigenetic diversity between sexes, and treatment outcomes of the disease. Some of these may stem from natural biological differences between the sexes, while others may result from sociocultural differences (e.g., lifestyle, access to healthcare) (reviewed in: [26,269]). Furthermore, epidemiological data suggest that obesity is associated with a 30–70% increased risk of CRC in men, but the association is less pronounced in women. In Europe, approximately 11% of CRC cases are attributed to overweight and obesity [270,271,272]. Other authors report that patients with a high BMI (>40 kg/m^2^) have a 20% or 1.18 times higher risk of developing CRC [273]. VO appears to be a more important risk factor for CRC than subcutaneous obesity. The exact mechanisms linking obesity to CRC are still being investigated and debated, but MetS, insulin resistance and changes in adipocytokine levels, inflammation, and changes in the gut microbiome appear to be the most important [247,271,272]. As research indicates, an increase in the abundance of pathogenic bacteria (*Escherichia coli*, *Butyricimonas virosa*, *Ruminococcus bicirculans*, *Bacteroides fragilis*, and *Streptococcus vestibularis*) is observed as CRC patients age. At the same time, the abundance of probiotics (*Eubacterium eligens*) decreased with the age of these patients. Therefore, the authors conclude that when using gut microbiota to predict CRC risk, age should be considered as a factor influencing the composition of the microbiota [274]. Further details on the role of the microbiota in colorectal carcinogenesis are provided in other review. Leading theories linking gut microbial dysbiosis with CRC are presented. Although SST plays a significant role in GI tract function, there is a lack of research on its direct interactions with the gut microbiome [275].

Although the relationship between obesity and the risk of CRC was confirmed in the latest systematic review from the USA, standardization of adiposity measurements is necessary [276]. The incidence of CRC in obese women and men has been explained by gender differences in the incidence and age of onset of MetS or the protective effect of estrogen, which may induce apoptosis and inhibit cell proliferation [270]. The latest meta-analysis also indicates a worse postoperative course in patients with CRC and VO, including a higher rate of conversion to open surgery [277]. Interestingly, a cross-sectional analysis of 163,129 individuals, conducted using data from the prospective database of the Polish Colonoscopy Screening Program, demonstrated that obese individuals had less advanced disease compared to non-obese individuals. Furthermore, survival by clinical stage did not appear to be influenced by BMI category. Similarly, overweight and obesity were not statistically significant predictors of mortality risk [278].

The role of age-related changes in SST expression in the pathogenesis of CRC is poorly understood and unclear. However, the role of SST as an antiproliferative and antisecretory peptide in the physiology and pathology of the GI tract has been proven. It has been demonstrated that SST inhibits colon cancer cell growth through cyclooxygenase-2 (Cox-2), prostaglandin E(2) (PGE(2)) production, DNA synthesis, and growth downregulation [279]. Other mechanisms of SST’s activity in CRC are presented in earlier reviews [27,39,135,275]. Studies also showed that poorly differentiated CRC exhibits a lower level of SST expression, whether SST is connected with metastasis of cancer is not established yet [27]. Recent study of Yue et al. provides molecular characterization of 15 hub age-related genes at the DNA and protein levels, as well as gene set enrichment analysis (GSEA) between high- and low-risk CRC groups. They indicate that the age-related signature is a reliable model for prognostic analysis and can predict the severity and immune cell infiltration in CRC patients. However, among the detected age-related hub genes there was no *SST* gene [280].

As indicated by a mouse model study, changes in SST concentration observed in various sections of the GI tract (including the colon) may be important for the increased gastrointestinal dysfunction in older adults. They may also contribute to the development of CRC [173]. In patients with CRC, disturbances in the LI neuroendocrine system also occur, which may influence tumor development. Colonic SST, galanin, and serotonin levels have been shown to be low in CRC patients [281,282]. The decrease in the number of SST cells and their CSI may explain the decreased levels of this peptide in the LI observed in these patients [281]. *SST* gene expression in CRC tissues and its correlations with patient age have been studied. A significant decrease in *SST* gene expression was demonstrated in CRC patients compared to samples collected from healthy adults or control tissues from the same patients [41,42]. However, studies by Leiszter et al. did not reveal changes in SST mRNA expression during normal aging (samples from healthy adults vs. samples from healthy adolescents) [41]. In contrast, our own studies showed a negative correlation of SST mRNA expression with age in both CRC and control groups [42].

It is well known that with age, GI tract motility disorders increase, which are regulated by enterohormones (including SST) [173,283,284]. A number of factors are considered in the pathogenesis of intestinal dysmotility, including (1) degeneration of enteric neurons and non-neuronal cell populations involved in GI tract motor function, (2) age-dependent metabolic and neuroendocrine changes, and (3) dietary factors (reviewed in [284]). Using IHC for study protein gene product 9.5 (PGP9.5) expression of age-matched mice, significantly fewer submucosal ganglia were observed in the colon of 1-Mo-old mice than 3-Mo-old mice. Furthermore, the colonic myenteric ganglia of 1-, 12-, and 24-Mo-old mice were smaller than those of 3-Mo-old mice. These changes may have some relevance to the increased GI tract motor dysfunction in older individuals [283]. Another study in mice indicates that aging of the ENS does not cause loss of myenteric neurons in the distal colon across the age groups studied, although neurodegenerative changes occur that may affect neuronal function [285]. In relation to human CRC, abnormal plasma enterohormone levels are significantly more common in colon cancer than in rectal cancer. Therefore, motility disorders observed in CRC patients may result from both anticancer surgery and abnormal enterohormone release due to the cancer itself or changes in the autonomic nervous system. However, this study did not concern the SST-ir nerves themselves [286].

Many studies show that CRC progression is accompanied by changes in the structure and plasticity of the ENS [287,288]. Gradual (depending on the degree of CRC invasion) partial or complete destruction and atrophy of neuronal elements in the ENS were demonstrated. A significant decrease in the number of neurons and nerve fibers was also observed in both the SP and MP. Interestingly, non-significant differences in the number of substance P(+) and SST(+) neurons were observed, as well as a similar density of SST(+) nerve fibers in the plexuses of intact versus pathologically changed areas [144].

The potential relationship between epigenetic changes of many genes (including *SST*) important in GI tract carcinogenesis in normal rectal biopsies and the presence or absence of coexisting LI adenomas, smoking status, and age was analyzed. In the study of adenomas alone, normalized methylation values for O-6 methylguanine-DNA methyltransferase (*MGMT*), retinoic acid receptor β (*RAR-β*), and *SST* showed a progressive decrease in the following order: nonsmokers without adenomas > smokers without adenomas > nonsmokers with adenomas > smokers with adenomas. A prognostic model for the presence of LI adenomas was developed based on gene methylation patterns in the normal rectum. The results indicate that these genes may be involved in the early stages of adenoma development. The epigenetic changes noted by the authors may be, at least in part, a result of smoking and/or advanced age [289].

Summary of studies on SST expression/level in different models of selected age-related chronic diseases of the GI tract (VO, T2DM, and CRC) is presented in Table 4.

In summary, the results of studies on SST expression depending on age concern various sections of the GI tract (stomach, duodenum, colon) and mostly note a decrease in SST expression/levels in EECs and ENS structures. However, the causal role of these changes in the development of age-related GI tract dysfunction is difficult to determine. It has been suggested that SST deficiency may be a pathogenic factor in obesity, although the mechanisms are poorly understood. There is a review also analyzing the problem of obesity and endocrine changes in aging men, but it lacks a detailed discussion of the role of the entire HPS axis (including SST) in this process. As noted, obesity and changes in body composition are the main consequences of somatopause/somatopenia in men, but there is a lack of comparison of these changes with those in aging women [64]. That, however, there is evidence suggesting that excessive postnatal feeding has a synergistic effect on the harmful effects of HFD-induced obesity on key components of the GH axis in adults, and the mechanisms differ between the two sexes [253].

In the pathogenesis of age-related T2DM in animal models, an age-dependent decrease in insulin mRNA levels has been confirmed, with no decrease in SST. In humans, however, lower SST pancreatic content has been shown in T2DM patients than in ND individuals, with unchanged SST/insulin and SST/glucagon ratios. Other studies have confirmed observations of a reduced number of δ cells in the pancreatic islets in T2DM. However, they have shown that T2DM is associated with excessive SST secretion, which may affect the paracrine regulation of insulin and glucagon secretion. There is a potential link between reduced SST expression and diabetic retinopathy, but this requires further study.

CRC is an example of a cancer in which age, male gender, obesity and alterations in the neuroendocrine system are important risk factors for tumor development. Lower levels of SST expression (mRNA, protein) were observed in CRC compared to controls, which may be due to a decrease in the number of SST(+) cells and their CSI in LI. As CRC progresses, the structure and plasticity of the ENS also change, including the loss of neurons and nerve fibers. In the elderly and in CRC, the composition of the gut microbiome also changes; however, the role of SST in controlling the colon microbiome remains undetermined.

**Figure 3:**
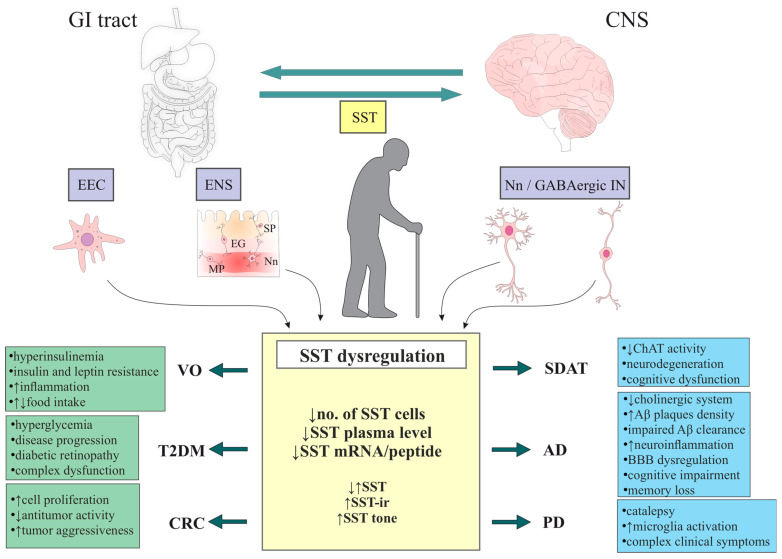
A diagram illustrating potential correlations between age-related changes in somatostatin expression/levels in the central nervous system (CNS) and gastrointestinal (GI) tract and the most common diseases in older adults affecting the CNS (e.g., Alzheimer’s disease and Parkinson’s disease) and the GI tract (e.g., obesity, diabetes mellitus, and colorectal cancer) [↑/↓—increase/decrease; Aβ—amyloid β peptide; AD—Alzheimer’s disease; BBB—blood–brain barrier; ChAT—choline acetyltransferase; CRC—colorectal cancer; EG—enteric glia; EEC—enteroendocrine cell; ENS—enteric nervous system; GABA—γ-aminobutyric acid; IN—interneuron; ir—immunoreactivity; no.—number; MP—myenteric plexus; Nn—neuron; PD—Parkinson’s disease; SDAT—senile dementia of the Alzheimer type; SP—submucosal plexus; SST—somatostatin; T2DM—type 2 diabetes mellitus; VO—visceral obesity].

A schematic of the potential correlation of the age-related changes in SST expression/level in the brain and GI tract and the most common diseases of advanced age is shown in Figure 3.

## 6. Somatostatin-Based Therapies in Age-Related Diseases

Due to the short half-life of SST (2–4 min) and its rapid degradation by peptidases, native SST is not useful as a therapeutic agent in clinical practice. Therefore, synthetic SST analogs (SSAs) have been developed, including octreotide (OCT), lanreotide (LAR), and pasireotide (PAS), which have prolonged the action of SST [5,10,290,291].

The use of therapy based on the mechanisms of action of SST is described in current excellent reviews [290,292,293]. The use of SSAs is the standard therapy for acromegaly and primarily highly differentiated NETs/NENs of the GI tract and pancreas, as well as other tumors that express SST receptors [291,294,295,296,297,298,299]. In older patients with advanced NETs, a potential survival benefit has been suggested with LAR administered within 1 year of diagnosis [300]. Current types of SST-based therapies for well-differentiated NETs in elderly patients are presented [35]. Furthermore, the three-dimensional structure of SST2, including the inactive/active SST2-G protein complex bound to different ligands, was presented, and Trp8-Lys9 (the W-K motif) in SSAs was shown to be a key motif stabilizing the lower part of the ligand-binding pocket. Activated SST2 inhibits hormone secretion and induces tumor cell apoptosis through various signaling pathways [299].

This subchapter will discuss therapeutic options based on the action of SST in relation to the selected diseases in elderly people analyzed in this paper.

SSAs were used in Alzheimer’s disease (AD) [211,221,301,302]. Initial use of OCT did not produce the expected clinical improvement [301]. In another study, relative memory improvement was observed in AD patients after OCT (Sandostatin) infusion and during hyperinsulinemia compared with placebo and hyperglycemia. Memory did not improve during hyperglycemia when insulin was inhibited by simultaneous OCT infusion [303]. The individual and interactive effects of insulin and OCT on memory and plasma GH levels in older adults were also examined. Watson et al. confirmed previous reports that insulin and OCT modulate memory in AD patients and indicated OCT-induced memory improvement in healthy older adults. This pioneering study also demonstrated that the apolipoprotein E ε4-negative (APOE-ε4(-)) genotype can modulate the effects of OCT on cognitive function [304].

In a mouse model, aging and gender were found to be important factors influencing behavioral parameters. Furthermore, SST4 was suggested to influence locomotion and exploratory behavior only in young mice, but not during normal aging. Therefore, the effect of SST4 agonists on locomotion and learning would be questionable [305]. However, recent preclinical studies in AD indicate that administration of SST or SST receptor agonists can improve cognitive function. The SST4 agonist (NNC 26-9100) has shown particular promise, potentially ameliorating learning and memory impairment, reducing comorbid symptoms, and improving enzymatic degradation of Aβ in the brain [211,306]. Recently, results were reported for a murine AD model overexpressing amyloid precursor protein (APP) with the Arctic mutation in Aβ. The therapy involved combining SST with a blood–brain barrier transporter and an Fc fragment to prolong the half-life of the complex. This strategy effectively reduced both oligomeric and larger Aβ aggregates, which may pose a challenge to other therapeutic approaches [302]. As indicated by the latest review, preclinical studies on the treatment of SSA in patients with AD suggest that this therapeutic approach may enhance amyloid clearance by activating neprilysin, alleviate tau pathology via the phosphoinositide 3-kinase/serine/threonine kinase Akt (PI3K/Akt) signaling pathway, regulate APOE4 expression, and modulate microglial function, thereby protecting synaptic integrity [307]. Although SST has significant therapeutic potential in the treatment of AD, it is not a standard treatment for the disease.

Aging and reduced SST activity in the brain are also key characteristics of PD. Experimental studies (rat model) have shown that exogenous SST, by directly acting on its specific receptors in the striatum, inhibits the effects of DA receptor activation in rats with parkinsonian symptoms. It was already suggested at that time that therapies based on SST modulation could prove effective in treating PD [308]. Santis et al. demonstrated the involvement of SST and two of its receptors (SST2 and SST4) in increasing locomotor activity in rats in a dose-dependent manner. These studies demonstrate that the activation of only some SST receptors in the striatum influences locomotor activity in rats, and that glutamatergic neurotransmission underlies SST-induced behaviors. SST increases glutamate release from corticostriatal terminals, and subsequent activation of excitatory glutamate receptors (AMPA/NMDA), likely located on DA terminals, is responsible for the SST-mediated increase in DA levels [309]. Another very interesting review specifies the role of SST as a modulator of presynaptic glutamate and noradrenergic signaling, explaining the main concepts of volume diffusion and metamodulation, which may contribute to the development of new forms of therapy for PD and other CNS diseases [310]. In a model of extrapyramidal dysfunction in PD, attempts were made to investigate whether inhibition of SST in the brain could induce catalepsy in young and aged rats. This motor impairment occurred in a dose-dependent manner after administration of cyclosomatostatin (cSST) (an SST antagonist) in aged rats, but not in young ones, and the effect was reversed by OCT. This study demonstrated for the first time that aging combined with SST deficiency in the brain could induce catalepsy in rats [236]. Although the results of SST in PD are promising, SSAs are not used in the standard treatment of PD.

SSA therapy also addresses obesity and diabetes mellitus—metabolic disorders that are often interrelated [90,311,312,313]. Because these are chronic diseases with significant heterogeneity, it was initially impossible to identify patient subgroups that would benefit most from SSA treatment [90]. Intravenous and intranasal administration of a long-acting SSA called L363,586 to people with diabetes effectively reduced both fasting and postprandial hyperglycemia. It also improved the glucose imbalance known as the dawn syndrome. Therefore, it was believed that this SSA, in combination with standard insulin therapy, might be useful in controlling unstable diabetes [311]. Other observations in T2DM have shown that OCT inhibits insulin and glucagon secretion, leaving glucose levels unchanged or slightly elevated. In T1DM, OCT reduces insulin requirements, but similarly to T2DM, GH inhibition was consistently observed only when the drug was administered before bedtime. However, hypoglycemia with OCT administration at night was problematic, as was its lack of effective GH inhibition (particularly in patients with proliferative retinopathy). Other drawbacks of OCT included a lack of specificity and gastrointestinal side effects, which opposed the clinical use of SSAs in diabetes at that time [312]. The next two randomized, controlled trials demonstrated that OCT delayed the progression of microvascular complications in preproliferative and advanced stages of diabetic retinopathy. Inhibition of early-phase insulin secretion with OCT in patients with hypothalamic obesity resulted in weight loss and improved quality of life. The efficacy of OCT correlated with residual β-cell activity before treatment [90]. Over the last dozen or so years, efforts have been underway to develop more universal ligands encompassing all known SST receptor molecules, as well as agents specific to receptor subtypes [314]. Thus, according to current views, potential therapeutic applications of SSAs include the treatment of diabetic complications such as retinopathy, nephropathy, and obesity, by inhibiting IGF-1, vascular endothelial growth factor (VEGF), along with insulin secretion and the effects on the renin–angiotensin–aldosterone system [293]. For the use of SSAs in diabetic retinopathy, it is necessary to further refine the selection of subgroups of patients who would benefit from such supplementation, and to better define the interactions of SST and its analogs administered exogenously with other retinal peptides in the context of hyperglycemia (reviewed in [315]). It should be added that diabetes is also common among patients with NETs of the GI tract (53% of the cohort). Despite previous studies suggesting an association between SSAs and hyperglycemia, the analysis by Ni et al. showed a similar risk of diabetes in patients with NETs treated with SSAs and those not treated with SSAs. However, research to understand this relationship should continue [316].

Molecular anti-obesity vaccines are also among the promising new targets, as described in the original paper [317] and in several review articles [313,318,319]. In a diet-induced obesity model in mice, the use of chimeric SST vaccines JH17 or JH18 via the intraperitoneal route was shown to result in significant weight loss compared to the control group. The observations regarding weight loss/lower weight gain were even more significant because all mice consumed similar amounts of food throughout the study. The presence of high levels of antibodies against SST after 6 wks correlated with the body weight observations and confirmed the efficacy of the vaccination [317].

In the case of CRC, SSAs have been shown to have anti-angiogenic and pro-apoptotic effects in human rectal NECs both in vitro [320] and in vivo [321]. Octreotide LAR significantly prolonged the time to tumor progression compared to placebo in patients with functionally active and inactive metastatic midgut NENs [322]. Radiolabeled SSA/peptide receptor radionuclide therapy (PRRT) has proven to be a breakthrough treatment method for patients with advanced GEP-NETs expressing SST receptors [296,321]. The most commonly used SSAs in clinical PRRT practice are OCT and octreotate (TATE). The latter demonstrates greater affinity and selectivity for SST2. Conjugation of both these peptides was performed with the bifunctional chelator 1,4,7,10-tetraazacyclododecane-1,4,7,10-tetraacetic acid (DOTA) to chelate ^177^Lu or ^90^Y [323,324]. Using the PRRT concept, Lutathera^®^ combines the radionuclide ^177^Lu with the SSA DOTA-TATE, thereby delivering ionizing radiation directly to cancer cells expressing SSTRs. Lutathera^®^ provokes DNA single- and double-strand breaks, in case of double-strand breaks leading to cell death of the tumor and its SSTR-positive lesions [325]. However, the challenge in NEN therapy is to determine the optimal forms of combination therapies, combining SSAs with chemotherapy and molecular therapies, and expanding the indications for SSAs therapy in other cancers [296].

In the treatment of sporadic CRC (primarily adenocarcinomas), SSA therapy has proven ineffective. A randomized, controlled, double-blind study of a large number of patients with advanced, asymptomatic CRC (*n* = 260) demonstrated that OCT administered at a dose of 150 micrograms three times daily was ineffective in reducing time to disease progression and survival [326]. Other authors who used OCT in patients with advanced gastrointestinal cancer (including 46 CRC patients) showed that patients treated with OCT had a significant survival advantage over controls (including CRC patients). Twenty-five patients (45%) receiving OCT had stable disease, compared with only eight (15%) in the control group. OCT therapy appears to provide a survival benefit in patients with chemotherapy-resistant advanced GI tract cancer. Additional studies are needed to confirm these results and clarify other issues regarding the dose and schedule of OCT [327].

SSAs have been used with good results in palliative therapy, primarily utilizing the antisecretory properties of SST [328,329,330]. A regimen of continuous infusion of OCT acetate at a dose of 150 micrograms/hour was an effective and safe treatment for chemotherapy-induced diarrhea combined with intestinal rest and intravenous fluid rehydration in a group of patients, most of whom were treated with a weekly regimen of 5-Fluorouracil (5-FU) and high-dose leucovorin [328]. Randomized controlled trials have confirmed the efficacy of OCT in the treatment of malignant intestinal obstruction and chemotherapy-induced diarrhea [330].

## 7. Limitations

The paper presents numerous examples of the coexistence between SST expression and age-related changes in physiology and diseases; however, a limitation is certainly the wide variety of research techniques used in studies from the 1980s and 1990s (hence the occasional discrepancies in results). There are not enough studies discussing the connection between animal research and its application to humans, and it is often impossible to establish actual cause-and-effect relationships in the described diseases. The length of this review does not allow me to cite all the studies; I acknowledge that the topic should be subjected to even more detailed analysis or a meta-analysis.

## 8. Concluding Remarks

The HPS axis is one of three endocrine systems in mammals that decline in hormone concentration during normal aging. Many studies over the past 30 years have focused on the process of somatopause, and GH deficiency has been considered a model of normal aging. The mechanisms of somatopause are very complex and multifactorial. One factor in the hypothalamic regulation of the HPS axis is undoubtedly SST, a hormone produced by both the CNS and the GI tract. The results of studies on SST expression in the CNS across age groups are not clear-cut, primarily due to the diversity of research models and techniques. These results also vary depending on the region of the CNS studied. Most studies on the rat hypothalamus show either a decrease in SST expression (mRNA and protein) or no change in SST expression with age. Recent transcriptomic studies of different types of human cortical cells during healthy aging have confirmed the decrease in the number of SST(+) and VIP(+)-IN observed in other research models, which has been considered the largest change in cortical cell proportions with age.

Lower levels of SST in the brain have been demonstrated in age-related CNS diseases such as SDAT, AD, and PD. In SDAT, there are degenerative changes in neurons and SST(+) fibers. In AD patients, the role of both reduced and hyperactive SST-IN function is emphasized. In AD, the level of selectively sensitive subtypes of SST-ir inhibitory neurons is reduced. A link between inhibitory neurons and resistance to AD pathology has also been discovered. In PD, significantly reduced levels of SST were observed in the frontal cortex, hippocampus, and entorhinal cortex in patients who suffered from mild to severe dementia. Some studies indicate a neuroprotective role for SST in PD and its involvement in inhibiting the loss of DA neurons in the substantia nigra.

Studies on SST expression in various sections of the GI tract (stomach, duodenum, colon) and in the pancreas have mostly shown a decrease in total SST levels with age. It has been suggested that age-related disturbances in SST expression play a role in the pathogenesis of numerous GI tract diseases in older people (e.g., obesity, T2DM, pancreatic and colon cancer). However, the exact mechanisms are unclear and require further research. It has been suggested that SST deficiency may be a pathogenic factor in obesity, although the mechanisms are poorly understood. Patients with T2DM have been shown to have fewer δ cells and lower total SST secretion in pancreatic islets than non-diabetic individuals. Studies (including transcriptomic analyses) suggest that increased pancreatic glucagon secretion and decreased total SST secretion contribute to hyperglycemia in patients with T2DM. Recent studies using real-time detection of SST release from individual pancreatic islets confirm the decrease in the number of δ cells in pancreatic islets in T2DM, but show an increase in SST secretion by isolated δ cells and their potential impact on the paracrine regulation of insulin and glucagon secretion in diabetes.

Significant risk factors for the development of CRC include advanced age, obesity (primarily visceral obesity), male gender, the presence of coexisting metabolic diseases (including T2DM)), and alcohol and tobacco use. Only for some of the listed risk factors (age, smoking) has a direct or indirect association with changes in SST expression in this type of cancer been demonstrated. In CRC, a decrease in serum levels and local SST expression (mRNA/protein) is observed, both in the EEC and in ENS structures. In CRC progression, numerous degenerative changes in ENS elements are found, which may account for the reduction in SST in these segments of the GI tract. However, based on the available studies, it is difficult to establish a cause-and-effect relationship for the observed changes. The role of SST in controlling the colon microbiome also remains undetermined.

The results of SST-based therapy in geriatric diseases have not yet been spectacular. The use of SSAs remains the gold standard for the treatment of acromegaly and NETs/NENs of various sites (primarily in GEP-NETs), where their role in stabilizing the disease has been confirmed. If SSAs are ineffective or cease to work, targeted radioisotope therapy using radioisotope-labeled SSAs is used. However, SST-based therapies are not widely used in geriatric diseases such as AD, SDAT, or PD, or in obesity and/or diabetes. SSAs have also been found to be clinically ineffective in sporadic CRC in vivo. However, SSAs are used with good results in palliative care, primarily for their antisecretory properties.

## Figures and Tables

**Table 1:** Summary of studies on SST expression/level in various central nervous system regions in animal models in relation to age.

Brain Area	Animal/Age	Method	Age-Related SST Expression/Level	Ref. No.
hypothalamus	Male Fisher 344 rats/3, 12, 20, 30 Mo	IHC	↓ SST	[110]
	Male Sprague-Dawley rats/3, 24 Mo	IHC	NS between two groups	[111]
	Male and female Wistar rats/10, 30, 75 days, 14 Mo	hypothalamic explants in vitro	↑ basal and K^+^-stimulated secretion of SST, NS in two sexes	[119] *
	Male Brown-Norway rats (ad libitum fed, and moderately calorically restricted male)/6, 16, 25 Mo	RIA	(i) ad libitum fed rats: ↓ GH secretory dynamics associated with ↓ total SST mRNA levels; (ii) ↑ SST mRNA precipitating with polyribosomes; (iii) ↑ polysomal/total mRNA levels in 25 vs. 6 or 16 Mo; (iv) moderate caloric-restricted rats: *NS* total SST mRNA or the polyribosome-associated SST mRNA vs. ad libitum fed 25 Mo	[127]
	Y (5 ± 0.3 yrs) and O (27 ± 0.7 yrs) female rhesus monkeys (*Macaca mulatta*)	PPP method, RIA	(i) ME: 2-fold ↑ mean SST levels during mornings and evenings in O vs. Y adults; (ii) > amplitude and baseline SST levels in O vs. Y; (iii) NS in the pulse frequency of SST release	[16]
	Male Wistar rats/3, 24 Mo	RIA; in vitro studies	(i) ↓ hypothalamic GHRH mRNA and ir GHRH in O rats, as did ME GHRH-ir; (ii) ↓ hypothalamic SST-ir and SST mRNA, and ↓ ME SST-ir in O rats as were GHS-R and IGF-1 mRNA	[120]
hypothalamus, CC	Male Fisher 344 rats/3–4, 12–14, 22 Mo	Northern blot analysis of RNA; ISH	(i) CC: NS in relative SST expression in all groups; (ii) hypothalamus: ↓ SST and SST mRNA/poly A + RNA ratios in O rats ab. 50% of levels in Y animals	[126]
	Rats/25 Mo	mRNA levels	(i) frontal CC: ↓ (−49%) pre-prosomatostatin mRNA; (ii) parietal cortex: ↓ (−80%); (iii) striatum: ↓ (−69%); (iv) hypothalamus: *NS*	[113]
hypothalamus, pituitary	Female and male Sprague-Dawley rats/adult, middle-aged and aging rats (17, 20, 27 Mo)	qISH	(i) PeVN: SST mRNA levels with sexual dimorphism in adult rats (males > females); (ii) ↓ SST mRNA in middle-aged and aging rats of both sexes, but NS among the three age groups	[117] *
	Female C57BL/6J mice/2, 4, 12, 23 Mo	IHC	(i) ↓ SST-ir Nn after 4 Mo; (ii) ↓ GH-ir cells from 2–4 Mo and 12–23 Mo as well as in size from 2–4 Mo and from 12–23 Mo; (iii) GHRH-ir Nn and SST-ir Nn < in O mice, SST/GHRH-ir-Nn ratio > in O mice	[124]
	Male C57BL/6J mice at 2, 4, 12, 24 Mo	IHC	(i) ↓ GHRH-ir Nn; (ii) NS SST-ir Nn between all groups; (iii) ↓ volume of the anterior pituitary and ↓ adenohypophysial parenchymal cells from 4–12 Mo; (iv) ↓ % and absolute number of GH-ir cells from 4–12 Mo and in size from 2–4 Mo and 4–12 Mo	[125]
	Male Sprague-Dawley rats/3, 12, 22 Mo		striatum and hypothalamus: ↓ SST and substance P in the day	[115]
	Male Wistar rats/2, 18–20 Mo	RIA	(i) ME: ↓ SST, peaked at the beginning of the activity phase (Y rats) or at the end of the rest phase (O rats); (ii) adenohypophysis: ↑ SST in O rats, peak values of advanced to early phases of rest span in O rats	[118]
pituitary	Male Fisher 344 rats/3–4, 12–14, 22–24 Mo	RIA	(i) ↓ basal GH release both with age and SST administration; (ii) in the presence of SST-14, GRF-induced release of GH < in O vs. Y or middle-aged rats	[112]
pituitary, CC	Male Wistar rats/90 days, 2 yrs	in vivo model of GH deficiency	(i) ↓ GH and IGF-1 levels and pituitary GH mRNA in 2-yr-O rats vs. adult rats; (ii) CC: ↓ SST-ir levels and SST- and IGF-1-mRNAs	[129]
hippocampus, striatum, CC, hypothalamus	Male Wistar rats/4, 18, 26 Mo	tissular protein levels	(i) hippocampus: ↑ SST levels; (ii) striatum: ↓ SST (4–18 Mo) and NS (18–26 Mo); (iii) frontal CC: NS between groups; (iv) hypothalamus: NS between groups	[114]
CC	SAMP8 mice model/4, 12 Mo	Microarray and RT-PCR	↓ SST expression	[106]
	Sprague-Dawley rats/4–6, 20–29 Mo	IHC	slight ↓ SST-ir cells in the parietal and occipital cortex	[116]
hippocampus, striatum	Two inbred C57BL and BALB/c Mice		(i) striatum: cholinergic activity and SST levels > disturbed in BALB/c vs. C57BL mice; (ii) hippocampus: NS SST and cholinergic neurotransmission	[121]
	Male Wistar rats/6, 18 Mo	effects of O-onset caloric restriction	(i) ↓ NPY- and SST-ir Nn with ↓ the cholinergic varicosities; (ii) 24 Mo caloric-restricted animals: NS number of those peptidergic Nn and the varicosities density vs. 12 Mo C rats	[122]
	Wistar rats/Y and O	ISH, IHC	(i) ↓ mRNAs of GABAergic IN molecular markers inversely correlated with ↑ of the α1 GABAR; (ii) SST and NPY mRNAs most frequently affected (75%); (iii) SST-IN more vulnerable than CALR-IN	[107]
	Male Wistar rats/3–4, 12, 24–26 Mo	RT and competitive PCR; IHC	(i) ↓ SST mRNA and SST-ir cells; (ii) inverse correlation between mRNA SST and IL-1β; (iii) ↓ SST-ir cells > in the hilus of dentate gyrus than in the CA1 region	[130]

Legend: ↑/↓—increase/high (upregulation/overexpression)/decrease/low (downregulation/lower expression); </>—lower/higher; *—data in both sexes; C—control; CA1—*cornu ammonis* 1; CALR—calretinin; CC—cerebral cortex; GABAR—γ-aminobutyric acid receptor; GH—growth hormone; GHRH/GRF—growth hormone-releasing hormone/factor; GHS-R—GH secretagogues-receptor; IHC—immunohistochemistry; IL-1β—interleukin 1β; IN—interneurons; ir—immunoreactive; K^+^—potassium ion; ME—median eminence; Mo—months; Nn—neurons; NPY—neuropeptide Y; NS—statistically nonsignificant; O—old/aged; PeVN—periventricular nucleus; poly A—polyadenylic acid; PPP—push–pull perfusion; qISH—quantitative in situ hybridization; Ref. No.—number of reference; RIA—radioimmunoassay; RT-PCR—(real-time)/reverse transcription-polymerase chain reaction; SAMP8 mice model—senescence-accelerated mice/prone 8 model; SST—somatostatin; SST-14—14-amino-acid form of SST; Y—young; yrs—years.

**Table 2:** Summary of studies on SST expression/level in different sections of the gastrointestinal tract in animal models in relation to age.

GI Tract Section	Animal/Age	Method	SST Expression/Level	Ref. No.
stomach	Rats	IHC	↓ G/SST cells ratio for the whole antrum from 6.5 at 7 d to 1.5 in old age	[169]
	Rats/4, 12, 24 Mo; bombesin, carbachol treatment		(i) ↓ basal level of SST; (ii) ↓ bombesin-stimulated release of SST in 12 and 24 Mo rats; (iii) inhibition of SST release by carbachol < in 24 Mo at 10^−8^ and 10^−7^ M, and < in 12 Mo rats at 10^−7^ M	[170]
	Mice/1, 3, 12, 24 Mo	IHC	(i) ↑ SST-ir cells in 1, 12, and 24 Mo vs. 3 Mo; (ii) ↑ CSI SST-ir cells in 1, 12, and 24 Mo vs. 3 Mo	[171]
	NMRI/Bom mice/1, 3, 12, 24 Mo	RIA	↓ SST level in 1, 12, 24 vs. 3 Mo	[173]
duodenum	NMRI/Bom mice/1, 3, 12, 24 Mo	RIA	↓ SST level in 1, 12, 24 vs. 3 Mo	[173]
	Mice/1, 3, 12, 24 Mo	IHC	(i) ↓ SST-ir cells in both the 12 and 24 Mo vs. 3 Mo; (ii) ↑ CSI of SST-ir cells in 12 Mo vs. 3 Mo and ↓ in 1 Mo vs. 3 Mo; (iii) ↓ nuclear volume of SST-ir cells in 12 Mo vs. 3 Mo	[172]
colon	Male Lobund–Wistar rats/2, 22, 28, 30, 33 Mo	IHC	NS for SST and chromogranin staining	[176]
	NMRI/Bom mice/1, 3, 12, 24 Mo	RIA	↓ SST level in 1 and 12 Mo vs. 3 Mo	[173]
pancreas	Fischer 344 rats/2, 10, 18, 24, 30 Mo; nutrient-induced, overnight-fasted		Only minor changes in SST release from the δ cells after the rat reached adulthood	[181]
	Rats/2–5, 24–27 Mo		(i) ↑ SST; (ii) ↑ sensitivity to SST’s inhibitory action	[182]
	Male Sprague-Dawley rats/Y, O and S	IHC	(i) pancreas histoarchitecture distortion in S animals; (ii) no abnormal changes in the non-B cell populations of the different groups	[183]
	Male mice (SAMP8/SAMR1)	RIA	NS for SST expression	[184]
small intestine	Rats/Y and O	EM	(i) ↓ SST-ir nerve fibers	[203]
	Sprague Dawley rats/Y and O	IHC	NS differences in SST-ir myenteric Nn	[204]
	Male Wistar rats/from newborn to senescence	IHC	(i) MP: ↑ % of SST-ir Nn during early postnatal development from 12 ± 2.4 in newborn rats to 23 ± 1.5 in 20 d, remaining stable until 60 d, ↓ % of SST-ir cells in 2 yr to 14 ± 2.0; (ii) SP: ↑ % of SST-ir Nn from 22 ± 3.2 in newborn rats to 42 ± 4.0 in 20 d, before ↓ in 2 yr to 21 ± 2.6	[205]
large intestine	Male Wistar rats/from newborn to senescence	IHC	(i) MP: ↑ % of SST-ir Nn during early postnatal development from 13 ± 3.0 in newborn rats to 18 ± 1.6 in 20 d, remaining stable until 60 d, ↓ % of SST-ir cells in 2 yr to 10 ± 2.6; (ii) SP: ↑ % of SST-ir Nn from 23 ± 1.7 in newborn rats to 32 ± 4.9 in 30 d, before ↓ in 2 yr to 28 ± 7.4	[205]

Legend: ↑/↓—increase/high (upregulation/overexpression)/decrease/low (downregulation/lower expression); <—lower level; d—days; CSI—cell secretory index; EM—electron microscope; G—gastrin; IHC—immunohistochemistry; ir—immunoreactive; Mo—months; MP—myenteric plexus; Nn—neurons; NS—statistically nonsignificant; O—old; Ref. No.—number of reference; RIA—radioimmunoassay; S—senescent; SAMP8—senescence-accelerated prone; SAMR1—senescence-accelerated resistant; SP—submucous plexus; SST—somatostatin; Y—young; yr—years.

**Table 3:** Somatostatin expression/level in different models of selected age-related diseases of the central nervous system: Alzheimer’s disease (AD), senile dementia of the Alzheimer’s type (SDAT) and Parkinson’s disease (PD).

Disease	Material/Model of the Study	Method	The Main Findings	Ref. No.
AD	Autopsied tissue of CC from AD pts	RIA	↓ SST-LI in CC	[224]
	Tissue from AD pts	IHC	SST-ir processes in neuritic plaques are present	[229]
	in vitro and in vivo paradigms		(i) SST regulates the metabolism of Aβ; (ii) SST-deficiency altered hippocampal neprilysin activity and localization, and ↑ the quantity of a Aβ42	[218]
	Female C and double-transgenic mice; 16, 30, 43, and 56 wks of age	(i) BrdU administration, perfusion, and sectioning; (ii) IF	(i) ↓ SST Nn with aging vs. C; (ii) Aβ aggregates with aging in the granule cell layer	[217]
	Temporal cortex and hippocampus of female and male AD and C	IHC	↓ SST-IN, but not PV-IN per mm^2^ in the temporal cortex vs. C subjects	[230]
	(i) Primary neuronal cells and neuroblastoma cells; (ii) Female and male C57B1/6 mice overexpressing the AβPP with the APPswe	(i) cell culture; (ii) ELISA (neprilysis and Aβ levels); (iii) SST-scFv8D3 administered to mice and its distribution studied by radiolabeling with iodine^125^	(i) after SST-scFv8D3 significant ↑ the brain concentration of neprilysin in APPswe mice; (ii) ↓ levels of membrane-bound Aβ42 in the hippocampus and the adjacent cortical area	[220]
	(i) All-atom molecular dynamics simulations of model mixtures of Aβ42 or Aβ40 peptides with SST-14; (ii) analysis of the structure and dynamics of early-stage aggregates		(i) Aβ42-SST-14 mixtures develop compact, dynamically stable, but small aggregates; (ii) SST-14 Ʇ aggregation in the Aβ42-SST-14 system despite a high hydrophobicity	[231]
	*App^NL-F/NL-F^* mice with SST-deficient mice	(i) genotyping; (ii) WB; (iii) neprilysin activity assay; (iv) IHC; (v) RT-PCR	(i) SST no impact on whole brain neprilysin transcript, protein or activity levels; (ii) SST-deficiency: ↑in the density of cortical Aβ plaques; (iii) SST interferes with early steps of Aβ assembly; (iv) no effect of SST on tau steady-state levels or its phosphorylation	[221]
	Human prefrontal cortex with cells from postmortem human brain samples from AD pts (*n* = 427)	a single-cell transcriptomic atlas	↓ selectively vulnerable SST inhibitory Nn subtypes	[228]
	A high-resolution cellular map of the aging neocortex of pts with various clinicopathology (*n* = 24)	(i) single-nucleus RNA sequencing; (ii) analytical method (CelMod); (iii) IHC; (iv) spatial transcriptomics	SST Nn subtype and oligodendroglial states among diverse cell populations associated with AD	[232]
	Autopsied tissues of entorhinal, occipito-temporal, dorsolateral prefrontal, and striate cortices with various stages of AD (*n* = 2)	multi-region spatial transcriptomics: IF	differences in the vulnerability of SST and SST chondrolectin inhibitory Nn and the expression of endosomal and lysosomal trafficking and metallothionein genes within the Aβ plaque microenvironment	[212]
SDAT	Postmortem brain (*n* = 15) and C tissue (*n* = 16)	IHC	↓ in ChAT activity in the temporal cortex accompanied by a ↓ (47%) in SST-ir	[225]
	Tissue of CC from SDAT pts	RIA	↓ SST-LI in CC	[224]
	Tissue form neocortex, C subjects	IHC; silver-impregnation method	(i) NS the density of SST-ir cells in the neocortex vs. aged C subjects; (ii) many SST fibers of abnormal morphology within senile plaques	[214]
PD	Both sexes of PD pts (*n* = 39) and C group (*n* = 39)	RIA	↓ SST (88.0 ± 4.1 pg/mL) vs. C (147.3 ± 5.1 pg/mL)	[234]
	cortex, hippocampus and caudate nucleus of PD pts	RIA	(i) ↓ SST in the frontal cortex in slightly or severely demented PD pts vs. C and non-demented Parkinsonians; (ii) ↓ SST in the hippocampus and entorhinal cortex of severely demented pts	[233]
	PD pts (*n* = 23) and subjects without neurological symptoms (*n* = 26)	RIA	↑ CSF-SLI content in PD pts (107.9 ± 9.8 pg/mL) vs. C subjects (73.5 ± 8.4 pg/mL)	[235]
	PD pts (*n* = 30), age-matched C (*n* = 10)	RIA	↑ mean value of serum SST at baseline in PD pts vs. C	[244]
	Model of extrapyramidal disorders in young and aged male Wistar rats	(i) bar test; (ii) intracerebroventricular administration of cSST	(i) cSST dose-dependently induced catalepsy in aged, but not in young rats; (ii) the cataleptic response reversed by a OCT	[236]
	Model of PD generated by injecting LPS into the SN of female (*n* = 144) Sprague Dawley rats	(i) IHC; (ii) WB; (iii) ELISA	(i) SST Ʇ the LPS-induced microglial activity and ROS production; (ii) ↓ TNF-α, IL-1β and PGE2 when SST applied prior to LPS; (iii) ↓ LPS-induced expression of iNOS, COX-2, and NF-κB p-p65 by supplying SST before LPS	[240]
	Male Wistar rats	(i) bar test; (ii) administration of cSST into the SN pars compacta (SNc), dorsal striatum (DS), locus coeruleus (LC), pedunculopontine tegmental nucleus (PPTg), and inferior colliculus (IC)	The intra-SNc, intra-DS, and intra-LC applied of cSST produced distinct cataleptic response	[237]
	*PARK2*-specific iPSCs derived from PD pts	(i) cell culture; (ii) IHC; (iii) qRT-PCR	(i) ↓ mRNA SST in GABAergic-IN derived from iPSCs of *PARK2*-specific PD pts as neural maturation progressed; (ii) ↓ SST(+) GABAergic Nn derived from iPSCs of pts with *PARK2* mutations	[241]
	Aged Wistar rats; 33 brain regions	intracerebroventricular injections of cSST	(i) ↓ c-Fos expression in the aged LEntCx; (ii) catalepsy in the aged rats probably associated with a ↓ of the LEntCx	[238]

Legend: ↑/↓—increase/high (upregulation/overexpression)/decrease/low (downregulation/hypoactivation/lower expression); Ʇ—inhibits; aa—amino acids; Aβ (40,42)—amyloid β peptide (40/42 aa); AβPP—Aβ-precursor protein; AD—Alzheimer’s disease; *App*—gene for amyloid precursor protein; APPswe—Swedish mutation; BrdU—bromodeoxyuridine; C—control(s); CC—cerebral cortex; c-Fos—proto-oncogene from Fos family; ChAT—choline acetyltransferase; COX-2—cyclooxygenase-2; CSF—cerebrospinal fluid; CSF-SLI—somatostatin-like immunoreactivity; cSST—cyclosomatostatin, SST antagonist; ELISA—enzyme-linked immunosorbent assay; GABA—γ-aminobutyric acid; IHC—immunohistochemistry; IF—immunofluorescence; IL-1β—interleukin 1 beta; IN—interneurons; iNOS—inducible nitric oxide synthase; iPSCs—induced pluripotent stem cells; ir—immunoreactive/immunoreactivity; LEntCx—lateral entorhinal cortex; LPS—lipopolysaccharide; *n*—number of pts; NF-κB—nuclear factor κB; Nn—neurons; NS—statistically nonsignificant; OCT—octreotide; *PARK2*—gene for Parkinson Protein 2 E3 Ubiquitin Protein Ligase; PGE2—prostaglandin E2; pts—patients; PV—parvalbumin; Ref. No.—number of reference; RIA—radioimmunoassay; ROS—reactive oxygen species; (q)RT-PCR—(quantitative) reverse transcription-polymerase chain reaction; scFv8D3—single-chain variable fragment of 8D3; SN—substantia nigra; SST—somatostatin; SST-14—14-aa form of SST; SST-scFv8D3—a recombinant fusion protein; SST-LI—SST-like immunoreactivity; TNF-α—tumor necrosis factor alpha; WB—Western blotting; wks—weeks.

**Table 4:** Somatostatin expression/level in different models of selected age-related chronic diseases of the GI tract: visceral obesity (VO), type-2 diabetes mellitus (T2DM), and colorectal cancer (CRC).

Disease	Material/Research Model	Method	The Main Findings	Ref. No.
VO	Adult male mice	(i) optogenetic experiments (stimulation of BF SST Nn, its projections to the LHA, and activation of BF VGAT Nn); (ii) behavioral studies; (iii) IF ISH	(i) ↑ fat and sucrose intake and ↑ anxiety-like behaviors; (ii) selective fat intake; (iii) ↑ general food intake and gnawing behaviors	[250]
	(i) Mice; (ii) Human female hypothalamus tissues (*n* = 2)	(i) *Gipr*-Cre knockin mice; (ii) primary culture of hypothalamic Nn; (iii) IHC; (iv) flow cytometry; (v) scRNA seq; (vi) qRT-PCR; (vii) calcium imaging; (viii) viral injections; (ix) food intake measurements; (x) RNAscope	(i) *Gipr* (+)expression in the hypothalamus, overlapped partially with *Glp1r* (humans and mice); (ii) *Gipr* cells included SST(+) Nn, glia, and vascular cells; (iii) activation of *Gipr* Nn using local AAV-delivered G_q_-DREADDs—↓ food intake	[251]
T2DM	Male Wistar rats/Newborn, 3, 6–7, 13–14, 23–24 Mo	(i) glucose oxidase method (Spectrum CCx); (ii) insulin (RIA); (iii) protein assay; (iv) isolation of pancreatic islets; (v) dispersion of pancreatic islets into single cells; (vi) RHPA; (vii) RT-PCR; (viii) slot-blot	(i) glucagon and SST mRNAs varied < vs. insulin in various age groups, showing that α- and δ-cells < affected by age; (ii) SST mRNA levels even more stable, and NS among groups	[259]
	C57BL/6J mice/3, 9, 30 Mo	slot-blot analysis	(i) SST mRNA levels unchanged; (ii) ↓ modesty of glucagon mRNA levels; (iii) 40% ↓ insulin mRNA with age	[260]
	Male and female ZDF rats/6–32 wks of age	IHC	(i) ↑ insulin and IAPP till 10 wks and thereafter ↓ rapidly; (ii) NS staining of glucagon, SST, and PP; (iii) even at the age of 32 wks, only β-cells are affected	[262]
	(i) Human diabetic postmortem eyes (*n* = 10) without clinically detectable retinopathy; (ii) eyes (*n* = 10) from ND donors	(i) RT-PCR; (ii) confocal laser; (iii) IF and WB; (iv) RIA; (v) TUNEL	(i) SST in RPE > neuroretina in both groups; (ii) SST mRNA and SST-28-ir in RPE/neuroretina from diabetic donors < ND donors; (iii) ↑ GFAP and ↑ degree of apoptosis in diabetic vs. ND retinas; these changes most evident in pts with > SST deficit	[268]
	Male ZDF and ZL control rats/9–13 wks or 30–34 wks	IHC	(i) NS % of SST(+) cells in young and elderly ZDF rats vs. ZL; (ii) NS % of insulin(+) cells in young, but ↓ in elderly ZDF rats vs. ZL; (iii) ↓ % of glucagon(+) cells in elderly ZDF rats and *NS* from ZL	[261]
	Autopsied human ND lean (*n* = 20) and obese (*n* = 19) and T2D (*n* = 18) pancreatic tissue samples	RIA	(i) SST level in T2D < ND; (ii) NS SST/insulin and SST/glucagon ratios; (iii) ↓ SST, insulin, and glucagon in T2D	[263]
	ND and T2D human pancreatic islet samples	(i) sc transcriptomes for 638 cells; (ii) bulk-cell RNA-seq; (iii) seq, read mapping, and quality C; (iv) sc sample processing and quality filtering; (v) islet cells classification/subpopulation analysis; (vi) differential gene expression analysis; (vii) gene set enrichment analysis; (viii) RNA ISH	(i) coexpression of LEPR in 77% of SST(+) cells in ND; (ii) δ cells express receptors from the leptin, ghrelin, and dopamine pathways; (iii) expression of genes linked to various forms of islet dysfunction/DM in δ and PP/γ cells	[265]
	(i) pancreatic islets from human cadaveric donors; (ii) mouse islets from 13–17-wk-old WT, ChR2^+/−^-SST^+/−^iCre or ChR2^+/−^-Glu^+/−^Cre	(i) islet isolation; (ii) IHC; (iii) cryosectioning; (iv) IF and confocal microscopy; (v) two-photon imaging; (vi) image analysis; (vii) direct-ELISA-based SST assay development; (viii) qPCR	(i) < δ-cells in the periphery of human vs. mouse islets; (ii) ↓ δ-cells in T2D donors vs. ND donors; (iii) ↓ δ-cell numbers and SST secretion in DM islets; (iv) ↓ SST mRNA expression in the human T2D islets	[264]
	(i) HeLa cells; (ii) pancreatic islets from C57Bl6J mice; (iii) human pancreatic islets from ND donors (*n* = 8) and T2DM donors (*n* = 4)	(i) plasmid construction; (ii) HeLa cell culture and transfection; (iii) islet isolation and culture; (iv) imaging of the cytoplasmic Ca^2+^concentration ([Ca^2+^]_i_) in islets and reporter cells; (v) IF and confocal imaging	(i) human T2DM pancreas: ↑ reporter cell [Ca^2+^]_i_ responses vs. ND islets, although < δ-cells identified; (ii) SST secretion regulated by glucose and highly sensitive to cAMP; (iii) ghrelin as a robust stimulator of SST secretion	[266]
CRC	Colon adenocarcinoma tissue samples (*n* = 12); C (*n* = 12)	IHC	(i) ↓ SST-ir and serotonin-ir cells in cancer; (ii) ↓ CSI of SST-ir and serotonin-ir cells in cancer; (iii) NS the nuclear volume of PP-, SST- and serotonin cells vs. C	[281]
	Tissue samples of CRC pts (*n* = 15) and C (*n* = 15)	IF	(i) decomposition and ↓ to final partial or complete destruction and absence of the Nn elements in cancer areas; (ii) NS in number of substance P(+) and SST(+) Nn; (iii) NS of density of SST(+) nerve fibers in the plexuses of C and cancer areas	[144]
	Normal rectal mucosal biopsies from a cohort pts who underwent serial colonoscopy by a single investigator (*n* = 113)	RT-q, methylation-specific PCR analysis	NMVs for MGMT, RAR-β, and SST: a gradual ↓ in the following sequence: nonsmokers without AD > smokers without AD > nonsmokers with AD > smokers with AD	[289]
	(i) colonic samples from healthy children (*n* = 6), healthy adults (*n* = 41) and CRC pts (*n* = 34); (ii) Caco-2 cell line	(i) SST mRNA expression analysis with HGU133 Plus2.0 microarrays; (ii) RT-PCR; (iii) IHC; (iv) OCT treatment of Caco-2 cells	(i) in normal aging: SST mRNA did not alter, but ↓ in cancer; (ii) ↑ the ratio of SST-ir cells in children vs. CRC; (iii) OCT—↑ % of apoptotic Caco-2 cells; (iv) ↑ SST methylation level in CRC vs. C young individuals	[53]
	(i) CRC (*n* = 34); and C (*n* = 33) pts; (ii) TMA with CRC (*n* = 33), lymph node metastases (*n* = 32) and C (*n* = 26)	(i) IHC; (ii) RT-qPCR	(i) ↓ SST (mRNA/peptide) and ↑ SST2 and SST5 (mRNA/peptide) in CRC vs. C; (ii) (−) correlation between SST mRNA and pts’s age in CRC and C	[54]

Legend: ↑/↓—increase/high (upregulation/overexpression/activation)/decrease/low (downregulation/reduced expression/hypoactivation); (+)/(−)—positive(ly)/negative(ly); </>—less, fewer, lower/higher; AAV—adeno-associated virus; AD—adenoma; BF—basal forebrain; C—control; cAMP—cyclic adenosine monophosphate; CSI—cell secretory index; CRC—colorectal cancer; DM—diabetes mellitus/diabetic; ELISA—enzyme-linked immunosorbent assay; GABA—γ-aminobutyric acid; GFAP—gial fibrillar acidic protein; *gipr*—gene for glucose-dependent insulinotropic polypeptide receptor G_q_—DREADDs-G_q_-designer receptors exclusively activated by designer drugs; IAPP—islet amyloid polypeptide; IF—immunofluorescence; IF ISH—immunofluorescent in situ hybridization; IHC—immunohistochemistry; ISH—in situ hybridization; LEPR—leptin receptor; LHA—lateral hypothalamic area; MGMT—O-6 methylguanine-DNA methyltransferase; Mo—months; *n*—number of patients; ND—non-diabetic; NMVs—normalized methylation values; Nn—neurons/neuronal; NS—statistically nonsignificant; OCT—octreotide; PP—pancreatic polypeptide; pts—patients; (q)RT-(q)PCR—(quantitative) reverse transcription/real-time polymerase chain reaction; RAR-β—retinoic acid receptor β; Ref. No.—number of reference; RHPA—reverse-hemolytic plaque assay; RIA—radioimmunoassay; RPE—retinal pigment epithelium; scRNAseq—single cell RNA sequencing; SST—somatostatin; SST-28—28-aa form of SST; T2D—type-2 diabetic; T2DM—type-2 diabetes mellitus; TMA—tissue microarray; TUNEL—transferase-mediated dUTP nick-end labeling; VGAT—GABAergic neurons; VO—visceral obesity; WB—Western blot; wk(s)—week(s); WT – wild type; ZDF—Zucker diabetic fatty; ZL—Zucker lean.

## Data Availability

No new data were created or analyzed in this study. Data sharing is not applicable to this article.

## Abbreviations

AA	amino acid
Aβ	amyloid β
AD	Alzheimer’s disease
AP	Parkinson’s disease
ARC	arcuate nucleus
CA1	*cornu ammonis* 1
cAMP	cyclic adenosine monophosphate
ChAT	choline acetyltransferase
CNS	central nervous system
COX-2	cyclooxygenase-2
CRC	colorectal cancer
CSI	cell secretory index
CST	cortistatin
EECs	enteroendocrine cells
EIF2	eukaryotic initiation factor 2
ENS	enteric nervous system
GABA	γ-aminobutyric acid
GCPGs	gangliocytic paragangliomas
GFAP	glial fibrillary acidic protein
GH	growth hormone
GHRH	growth hormone-releasing hormone
GIP	gastric inhibitory peptide
GI tract	gastrointestinal tract
GLP-1	glucagon-like peptide-1
GPCR	G protein-coupled receptors
G_q_-DREADDs	G_q_-designer receptors exclusively activated by designer drugs
5-HT	5-hydroxytryptamine (serotonin)
IGF-1	insulin-like growth factor-1
IHC	immunocytochemical/immunohistochemistry
iNOS	inducible NO synthase
LI	large intestine
MAPK	mitogen-activated protein kinase (originally called ERK)
ME	median eminence
Mo	month/months
MP	myenteric plexus/plexuses
mTOR	mammalian target of rapamycin
NAD(+)	nicotinamide adenine dinucleotide(+)
NEC(s)	neuroendocrine cell(s)
NEN(s)	neuroendocrine neoplasm(s)
NET(s)	neuroendocrine tumor(s)
NF-κB	nuclear factor kappa-light-chain-enhancer of activated B cells
nNOS	nitric oxide synthase
NP	neuropeptide/neuropeptides
panNET/pNET	pancreatic NET
PARK2	parkin RBR E3 ubiquitin protein ligase
PeVN	periventricular nucleus
PNS	peripheral nervous system
PP	pancreatic polypeptide
PVN	paraventricular nucleus
SASP	senescence-associated secretory phenotype
SDAT	senile dementia of the Alzheimer type
SI	small intestine
SP	submucosal plexus
SRIF/SRIH/SST	somatotropin-release inhibitory factor/hormone/somatostatin
SSAs	somatostatin analogs
T2DM	type 2 diabetes mellitus
TFF2	cytoprotective trefoil factor 2
VIP	vasoactive intestinal peptide
VO	visceral obesity
